# Contribution of malnutrition to infant and child deaths in Sub-Saharan Africa and South Asia

**DOI:** 10.1136/bmjgh-2024-017262

**Published:** 2024-12-05

**Authors:** Zachary J Madewell, Adama Mamby Keita, Priya Mehta-Gupta Das, Ashka Mehta, Victor Akelo, Ogony Benard Oluoch, Richard Omore, Dickens Onyango, Caleb K Sagam, Carrie Jo Cain, Cornell Chukwuegbo, Erick Kaluma, Ronita Luke, Ikechukwu Udo Ogbuanu, Quique Bassat, Milton Kincardett, Inacio Mandomando, Natalia Rakislova, Rosauro Varo, Elisio G Xerinda, Ziyaad Dangor, Jeanie du Toit, Sanjay G Lala, Shabir A Madhi, Sana Mahtab, Markus Roos Breines, Ketema Degefa, Helina Heluf, Lola Madrid, J. Anthony G Scott, Samba O Sow, Milagritos D Tapia, Shams El Arifeen, Emily S Gurley, Mohammad Zahid Hossain, Kazi Munisul Islam, Afruna Rahman, Portia C Mutevedzi, Cynthia G Whitney, Dianna M Blau, Parminder S Suchdev, Karen L Kotloff, The Child Health and Mortality Prevention Surveillance Network, Fatima Solomon

**Affiliations:** 1 Global Health Center, Centers for Disease Control and Prevention, Atlanta, Georgia, USA; 2 Centre pour le Développement des Vaccins, Ministère de la Santé, Bamako, Mali; 3 Rollins School of Public Health, Emory University, Atlanta, Georgia, USA; 4 Center for Vaccine Development and Global Health, University of Maryland School of Medicine, Baltimore, Maryland, USA; 5 US Centers for Disease Control and Prevention, Kisumu, Kenya; 6 Kenya Medical Research Institute, Nairobi, Nairobi County, Kenya; 7 Kisumu County Department of Health, Kisumu, Nyanza, Kenya; 8 World Hope International, Freetown, Sierra Leone; 9 Federal Medical Centre, Umuahia, Nigeria; 10 Crown Agents, Freetown, Sierra Leone; 11 Ministry of Health and Sanitation, Freetown, Sierra Leone; 12 ISGlobal–Hospital Clínic, Universitat de Barcelona, Barcelona, Spain; 13 Centro de Investigação em Saúde de Manhiça–CISM, Manhica, Maputo, Mozambique; 14 Hospital Sant Joan de Déu, Universitat de Barcelona, Barcelona, Spain; 15 ICREA, Pg. Lluís Companys 23, Barcelona, Spain; 16 Consorcio de Investigación Biomédica en Red de Epidemiología y Salud Pública–CIBERESP, Madrid, Spain; 17 Instituto Nacional de Saúde, Ministério de Saúde, Maputo, Mozambique; 18 South African Medical Research Council Vaccines and Infectious Diseases Analytics Research Unit, University of the Witwatersrand, Johannesburg, South Africa; 19 Department of Paediatrics & Child Health, University of the Witwatersrand, Johannesburg, South Africa; 20 Wits Infectious Diseases and Oncology Research Institute, University of the Witwatersrand, Johannesburg, South Africa; 21 Department of Infectious Disease Epidemiology, London School of Hygiene & Tropical Medicine, London, UK; 22 College of Health and Medical Sciences, Haramaya University, Dire Dawa, Ethiopia; 23 International Centre for Diarrhoeal Disease Research Bangladesh, Dhaka, Bangladesh; 24 Department of Epidemiology, Johns Hopkins Bloomberg School of Public Health, Baltimore, Maryland, USA; 25 Emory Global Health Institute, Emory University, Atlanta, Georgia, USA; 26 Department of Pediatrics, Emory University, Atlanta, Georgia, USA

**Keywords:** Global Health, Child health, Epidemiology, Nutrition, Paediatrics

## Abstract

**Introduction:**

Malnutrition contributes to 45% of all childhood deaths globally, but these modelled estimates lack direct measurements in countries with high malnutrition and under-5 mortality rates. We investigated malnutrition’s role in infant and child deaths in the Child Health and Mortality Prevention Surveillance (CHAMPS) network.

**Methods:**

We analysed CHAMPS data from seven sites (Bangladesh, Ethiopia, Kenya, Mali, Mozambique, Sierra Leone and South Africa) collected between 2016 and 2023. An expert panel assessed each death to determine whether malnutrition was an underlying, antecedent or immediate cause or other significant condition. Malnutrition was further classified based on postmortem anthropometry using WHO growth standards for underweight (z-scores for weight-for-age <−2), stunting (length-for-age <−2), and wasting (weight-for-length or MUAC Z-scores <−2).

**Results:**

Of 1601 infant and child deaths, malnutrition was considered a causal or significant condition in 632 (39.5%) cases, including 85 (13.4%) with HIV infection. Postmortem measurements indicated 90.1%, 61.2% and 94.1% of these cases were underweight, stunted and wasted, respectively. Most malnutrition-related deaths (n=632) had an infectious cause (89.1%). The adjusted odds of having malnutrition as causal or significant condition were 2.4 (95% CI 1.7 to 3.2) times higher for deaths involving infectious diseases compared with other causes. Common pathogens in the causal pathway for malnutrition-related deaths included *Klebsiella pneumoniae* (30.4%), *Streptococcus pneumoniae* (21.5%), *Plasmodium falciparum* (18.7%) and *Escherichia coli/Shigella* (17.2%).

**Conclusion:**

Malnutrition was identified as a causal or significant factor in 39.5% of under-5 deaths in the CHAMPS network, often in combination with infectious diseases. These findings highlight the need for integrated interventions addressing both malnutrition and infectious diseases to effectively reduce under-5 mortality.

**WHAT IS ALREADY KNOWN ON THIS TOPIC**

- Malnutrition, particularly undernutrition characterised by underweight, stunting and wasting, is a significant contributor to child mortality in low and middle-income countries (LMICs). Previous estimates have suggested that malnutrition is involved in 45% of under-5 deaths globally, with a substantial burden in Africa and Asia. However, the exact mechanisms by which malnutrition contributes to death and its interaction with infectious diseases remain incompletely understood.

**WHAT THIS STUDY ADDS**

- This study provides detailed postmortem data from seven LMICs, revealing that malnutrition plays a significant role in the causal chain of 40% of deaths among children under 5 years of age, either as the underlying cause or as a significant contributing factor. The study also highlights the high prevalence of severe malnutrition among deceased children, particularly in cases involving infectious diseases such as lower respiratory infections, sepsis and diarrheal diseases.

**HOW THIS STUDY MIGHT AFFECT RESEARCH, PRACTICE OR POLICY**

- The findings from this study underscore the urgent need for integrated approaches that combine nutritional support with effective prevention and treatment of infectious diseases in children. This study advocates for the implementation of comprehensive child health strategies in LMICs that address the dual burden of malnutrition and infection, with a focus on early detection and intervention to reduce child mortality. The evidence provided may also inform future research on the pathways linking malnutrition to fatal outcomes, guiding policy decisions and resource allocation for child health programmes.

## Introduction

 Despite declines during the past decade in the estimated prevalence of malnutrition among young children in low and middle-income countries (LMIC), a large burden continues to threaten child survival and well-being. Herein we describe the form of malnutrition termed ‘undernutrition’, which is typically defined anthropometrically as underweight, wasting and/or stunting. Global agencies estimated that in 2022, 22% of children under 5 years were stunted and 7% wasted, with children in Asia and Africa accounting for >90% of global burden.^1^ Each deficit has been associated with an increased risk of all-cause under-5 mortality .^2^,^4^

In the past decade, recognition of uncertainties about the mechanisms by which malnutrition leads to death has prompted a re-examination of paradigms depicting the role of malnutrition in <5 mortality, whereas older studies focused on the role of each deficit individually, recent analyses suggest that stunting, wasting and underweight share aetiologies and are correlated often with a multiplicative effect on mortality.^23 5^,^7^

Because death is a rare event, prospective longitudinal studies have been unable to clearly elucidate the underlying role of malnutrition and its specific forms in the causal chain that led to death. Assessments as to whether malnutrition contributed to the underlying cause of death, the immediate cause of death and the potential contributions of comorbidities such as infection and immunodeficiency are poorly understood. Modelled estimates suggest that undernutrition contributes to 45% of under-5 deaths,^8^ but caution is warranted in interpreting these projections due to inherent uncertainties, assumptions and a lack of sufficient information to understand the complete causal chain of events.^9 10^

The multinational Child Health and Mortality Prevention Surveillance (CHAMPS) network was created to address gaps in understanding the causes of mortality among children younger than 5 years of age in Africa and South Asia.^11^,^13^ CHAMPS provides a comprehensive evaluation of causes of death (CoD) by assessing a compilation of data derived from standardised clinical, epidemiologic and laboratory procedures. In this paper, we report the prevalence of malnutrition from postmortem examination, assess its role in the causal chain of events leading to death and examine links between malnutrition and infections in the CoD among deceased infants and children aged 1–59 months enrolled in CHAMPS. Children with and without HIV infection are included in our analysis, recognising that while malnutrition in the face of HIV may have unique medical and social pathogenetic mechanisms, HIV-infected and exposed children living in low resource settings with food insecurity face similar challenges to their uninfected peers.^14^

## Methods

### Study design

We analysed CHAMPS data collected at seven CHAMPS sites (Bangladesh, Ethiopia, Kenya, Mali, Mozambique, Sierra Leone and South Africa) between December 2016 and December 2023. Standardised data for each decedent were derived from clinical chart review, verbal autopsy, postmortem physical examination, anthropometry, photographs and blood culture, as described in detail elsewhere.^15^,^17^ Biopsy specimens obtained using minimally invasive tissue sampling (MITS) were examined for organ system-specific pathogens using quantitative PCR and histopathology. An expert Determination of Cause of Death (DeCoDe) panel adjudicated the data from each participant to determine causal chain leading to death, which includes a single underlying and immediate cause and any associated morbid conditions.^11 12^ Pathogen causality was rigorously assigned by the DeCoDe panels, who attributed specific pathogens to diseases such as pneumonia or sepsis, following standardised diagnostic criteria developed by CHAMPS.^18^ We included all infants and children aged 1–59 months who enrolled in CHAMPS, had MITS performed and had a cause of death assigned by the DeCoDe process. Our study followed Strengthening the Reporting of Observational Studies in Epidemiology reporting guidelines for cross-sectional studies.

### Malnutrition

Length, weight and mid-upper arm circumference (MUAC) were measured postmortem by trained study staff during the MITS procedure using calibrated equipment when possible.^19^ WHO Child Growth Standards were used to calculate z-scores for weight-for-age (WAZ) as a measure of underweight, length-for-age (LAZ) as a measure of stunting (chronic malnutrition) and both weight-for-length (WLZ) and MUAC Z-scores (MUACZ) as a measure of wasting (acute malnutrition).^20 21^ Z-scores were categorised as: normal (≥−2), moderate (−3 ≤ and <−2), and severe (<−3) and further characterised as any moderate-to-severe malnutrition (WAZ or LAZ or WLZ or MUACZ <−2) and any severe malnutrition (WAZ or LAZ or WLZ or MUACZ <−3). While some diagnostic criteria for severe acute malnutrition in children 6–59 months include a fixed MUAC cut-off of <11.5 cm, we elected to use either WLZ<−2 or MUACZ <−2 to define wasting as meta-analyses have shown that each measure confers a similar mortality risk.^22^ We excluded implausible anthropometric values (WAZ <−10 or >5, LAZ <−10 or >6, WLZ <−10 or >5, or MUACZ <−10 or >5), using a lower threshold than standard Z-scores for living children^23^ to account for changes in weight caused by the child’s fatal illness or any desiccation.

Malnutrition was included in the causal chain in this analysis only when deemed to be there by DeCoDe panellists. Therefore, not all children with Z-scores meeting criteria for malnutrition were considered to have malnutrition in the causal chain. Malnutrition-related deaths were defined as either: (1) deaths for which DeCoDe panels listed malnutrition (ICD-10 codes: E40–E46 or ICD-11 codes: 5B50–5B54, 5B7Y, 5B7Z) or HIV-related wasting syndrome (ICD-10 code: B22.2) in the causal chain or (2) deaths in which DeCoDe panels considered malnutrition as ‘other significant’, meaning that it may have contributed to death but was not a necessary step in the causal chain. In the ICD-11 coding system, HIV-related wasting is represented by separate codes for HIV (1C62) and malnutrition (eg, 5B50–5B54), reflecting a shift from the single code (B22.2) used in ICD-10. HIV infection status was determined by testing postmortem blood samples for HIV DNA or RNA using PCR. ICD codes began transitioning from ICD-10 to ICD-11 across CHAMPS sites during 2022–2023.

### Statistical analysis

Statistical analyses were performed using R software, V.4.4.0 (R Foundation for Statistical Computing, Vienna, Austria) and are described in detail in online supplemental eMethods. We reported descriptive statistics of anthropometric characteristics, ICD-10 codes, CoD, pathogens, coinfections and preventability of deaths with malnutrition in the causal chain or as another significant condition. We used mixed-effect logistic regression to assess associations between malnutrition and different CoD (eg, sepsis, lower respiratory infections), adjusting for age group, sex and death location as fixed effects, and site as a random effect. Furthermore, we evaluated associations between malnutrition and any infectious disease in the causal chain (congenital infection, lower respiratory infections, diarrheal diseases with an identified etiologic agent, malaria, measles, meningitis/encephalitis, other infections, rabies, sepsis, syphilis, tuberculosis or upper respiratory infections).

## Results

Between December 2016 and December 2023, 4382 infant and child deaths were identified by the CHAMPS team. Among the 2086 (47.6%) cases for which consent was obtained, 2072 (99.3%) underwent MITS. Results from the 1601 (77.3%) that completed DeCoDe adjudication are reported herein (online supplemental eFigure 1). The number of enrolled under-5 deaths by CHAMPS site is shown in online supplemental eFigure 2, with 272, 799, 630, 487, 671, 582 and 421 deaths from Bangladesh, Ethiopia, Kenya, Mali, Mozambique, Sierra Leone and South Africa, respectively. Of these, 10, 89, 379, 133, 261, 375 and 354 underwent MITS and were included in this analysis, respectively.

Malnutrition was included in the causal chain of 493 (30.8%) of the 1601 deaths and was considered the underlying CoD in 376 (76.3%), the antecedent CoD in 118 (23.9%) and the immediate CoD in 2 (0.4%) (table 1). In 141 (8.8%) deaths, malnutrition was coded as an ‘other significant’ condition. In total, 632 decedents had malnutrition either in the causal chain or listed as another significant condition; seven deaths had malnutrition listed two times in either the causal chain or as other significant condition (table 1). There were 573 (35.8%) other deaths that met the anthropometric criteria for malnutrition but were not cited as causal or significant condition by the DeCoDe panel (table 2). In total, 1188 (74.2%) of the 1601 deaths met anthropometric criteria for moderate-to-severe malnutrition of whom 908 (76.4%) were considered severe (table 1, online supplemental eTable 1). Anthropometric criteria for moderate-to-severe malnutrition were seen in nearly all deaths considered malnutrition-related (615/632, 97.5%) but also in many children (573/969, 59.2%) whose deaths were not considered related (table 2, online supplemental eTable 1). By comparison, clinicians documented malnutrition in the clinical chart antemortem in 41.7% of deaths overall and in 68.2% of those deemed by the DeCoDe panel to be causal or significant (table 2).

**Table 1:** Cause-of-death classifications and ICD codes* assigned by CHAMPS Determination of Cause of Death (DeCoDe) process for infant and child deaths with malnutrition in causal chain or as other significant condition by age group, CHAMPS, 2016–2023

Malnutrition classified as:	Overall	1–5 months	6–11 months	12–23 months	24–59 months
	N=632†	N=148	N=159	N=198	N=127
Anywhere in causal chain	493 (78.0)	107 (72.3)	127 (79.9)	166 (83.8)	93 (73.2)
Underlying cause of death	376 (59.5)	68 (45.9)	108 (67.9)	137 (69.2)	63 (49.6)
Immediate cause of death	2 (0.3)	0 (0)	1 (0.6)	0 (0)	1 (0.8)
Antecedent cause of death	118 (18.7)	39 (26.4)	19 (11.9)	31 (15.7)	29 (22.8)
Other significant condition	141 (22.3)	42 (28.4)	32 (20.1)	33 (16.7)	34 (26.8)
Assigned malnutrition-related ICD-10 codes:					
E40: Kwashiorkor	49 (9.2)	8 (6.7)	12 (8.8)	17 (9.8)	12 (11.3)
E41: Nutritional marasmus	191 (35.7)	50 (41.7)	48 (35.3)	64 (37.0)	29 (27.4)
E42: Marasmic kwashiorkor	71 (13.3)	8 (6.7)	17 (12.5)	28 (16.2)	18 (17.0)
E43: Unspecified severe protein-calorie malnutrition	52 (9.7)	18 (15.0)	15 (11.0)	13 (7.5)	6 (5.7)
E44: Protein-calorie malnutrition of moderate and mild degree	35 (6.5)	3 (2.5)	12 (8.8)	9 (5.2)	11 (10.4)
E44.0: Moderate protein-energy malnutrition	46 (8.6)	7 (5.8)	17 (12.5)	13 (7.5)	9 (8.5)
E44.1: Mild protein-energy malnutrition	1 (0.2)	0 (0)	0 (0)	0 (0)	1 (0.9)
E45: Retarded development following protein-calorie malnutrition	12 (2.2)	4 (3.3)	3 (2.2)	1 (0.6)	4 (3.8)
E46: Unspecified protein-calorie malnutrition	31 (5.8)	16 (13.3)	4 (2.9)	6 (3.5)	5 (4.7)
B22.2: HIV disease resulting in wasting syndrome	54 (10.1)	7 (5.8)	9 (6.6)	25 (14.5)	13 (12.3)
Assigned malnutrition-related ICD-11 codes:					
5B50: Underweight in infants, children or adolescents	1 (0.2)	0 (0)	0 (0)	0 (0)	1 (0.8)
5B51: Wasting in infants, children or adolescents	11 (1.7)	1 (0.7)	3 (1.9)	5 (2.5)	2 (1.6)
5B52: Acute malnutrition in infants, children or adolescents	31 (4.9)	11 (7.4)	4 (2.5)	9 (4.5)	7 (5.5)
5B7Z: Unspecified undernutrition	3 (0.5)	0 (0)	0 (0)	2 (1.0)	1 (0.8)

* ICD codes began transitioning from ICD-10 to ICD-11 across CHAMPS sites in 2022–2023, which is why both classifications are presented.

† There were 632 deaths with malnutrition as causal or significant condition: Seven deaths had malnutrition listed twice in either the causal chain or as other significant condition with different ICD-10 codes: three as underlying and antecedent cause, one as underlying and significant condition, one as antecedent and significant condition, one as antecedent cause twice, and one as significant condition twice.

**Table 2:** Characteristics of infant and child deaths with and without malnutrition in causal chain or as other significant condition, CHAMPS, 2016–2023

Characteristic	Overall	With malnutrition	Without malnutrition	P value
	N=1601	N=632	N=969	
Age in days (median (IQR))	335 (130, 709)	378 (195, 651)	300 (104, 758)	0.006
Age group				<0.001
1–5 months	518 (32.4)	148 (23.4)	370 (38.2)	
6–11 months	319 (19.9)	159 (25.2)	160 (16.5)	
12–23 months	380 (23.7)	198 (31.3)	182 (18.8)	
24–59 months	384 (24.0)	127 (20.1)	257 (26.5)	
Sex (%) (N=1600)				0.702
Female	726 (45.4)	291 (46.0)	435 (44.9)	
Male	874 (54.6)	341 (54.0)	533 (55.1)	
Location of death				0.004
Community	459 (28.7)	207 (32.8)	252 (26.0)	
Facility	1142 (71.3)	425 (67.2)	717 (74.0)	
Hospital duration in hours (median (IQR)) (N=948)	30 (9, 153)	41 (13, 142)	24 (8, 177)	0.051
Site				<0.001
Bangladesh	10 (0.6)	4 (0.6)	6 (0.6)	
Ethiopia	89 (5.6)	78 (12.3)	11 (1.1)	
Kenya	379 (23.7)	164 (25.9)	215 (22.2)	
Mali	133 (8.3)	58 (9.2)	75 (7.7)	
Mozambique	261 (16.3)	105 (16.6)	156 (16.1)	
Sierra Leone	375 (23.4)	157 (24.8)	218 (22.5)	
South Africa	354 (22.1)	66 (10.4)	288 (29.7)	
Time from death to MITS in hours (median (IQR))	13 (6, 21)	11 (4, 18)	14 (7 23)	<0.001
HIV status (%)				<0.001
Uninfected or unknown	1287 (80.4)	489 (77.4)	798 (82.4)	
Exposed uninfected	173 (10.8)	58 (9.2)	115 (11.9)	
Infected	141 (8.8)	85 (13.4)	56 (5.8)	
Antemortem diagnosis from clinical record (%)				
Any malnutrition	668 (41.7)	431 (68.2)	237 (24.5)	<0.001
Marasmus	233 (14.6)	185 (29.3)	48 (5.0)	<0.001
Kwashiorkor	56 (3.5)	46 (7.3)	10 (1.0)	<0.001
Count of causal conditions identified (%)				<0.001
No condition identified by Decode panel	57 (3.6)	5 (0.8)	52 (5.4)	
1	429 (26.8)	70 (11.1)	359 (37.1)	
2	459 (28.7)	168 (26.6)	291 (30.1)	
3	346 (21.6)	200 (31.6)	146 (15.1)	
≥4	309 (19.3)	189 (29.9)	120 (12.4)	
Median (IQR)	2 (1 3)	3 (2 4)	2 (1 3)	
Birth weight (%) (N=527)				<0.001
Extremely low birth weight	17 (3.2)	0 (0.0)	17 (5.1)	
Very low birth weight	34 (6.5)	3 (1.5)	31 (9.3)	
Low birth weight	90 (17.1)	35 (18.0)	55 (16.5)	
Normal weight	373 (70.8)	153 (78.9)	220 (66.1)	
Macrosomia	13 (2.5)	3 (1.5)	10 (3.0)	
Weight at MITS (kg) (median (IQR)) (N=1583)	6.5 (4.4, 9.1)	5.7 (4.2, 7.3)	7.6 (4.6, 10.5)	<0.001
Any moderate to severe malnutrition by post-mortem measurements (WAZ<-2 or LAZ<-2 or WLZ<-2 or MUACZ<-2) (%)	1188 (74.3)	615 (97.5)	573 (59.2)	<0.001
Any severe malnutrition by post-mortem measurements (WAZ<-3 or LAZ<-3 or WLZ<-3 or MUACZ<-3) (%)	908 (56.8)	537 (85.1)	371 (38.3)	<0.001
Deemed preventable or preventable under certain conditions from DeCoDe panel (%) (n=1553)	1321 (85.1)	567 (92.0)	754 (80.5)	<0.001

DeCoDe, Determination of Cause of Death panel; IQR, interquartile range; LAZ, length-for-age Z-score; MITS, minimally invasive tissue sampling; MUACZ, mid-upper arm circumference Z-score; WAZ, weight-for-age Z-score; WLZ, weight-for-length Z-score.

Among the 1601 deaths, 54.6% were men, 71.3% died in healthcare facilities and 28.7% died in the community (table 2); a similar distribution was seen among children with malnutrition in the causal chain or considered ‘other significant’ (online supplemental eTable 2). Children with malnutrition-related death were older than children without malnutrition in the causal pathway (378 vs 300 days, p=0.006); there were no differences by sex (p=0.702). The proportion of deaths deemed to be malnutrition-related was highest in Ethiopia (87.6%, 78/89), followed by Mali (43.6%, 58/133), Kenya (43.3%, 164/379), Sierra Leone (41.9%, 157/375), Mozambique (40.2%, 105/261), Bangladesh (40.0%, 4/10) and South Africa (18.6%, 66/354) (table 2, online supplemental eTable 3).

HIV-infection was identified in 141 (8.8%) of the 1601 adjudicated deaths, including 85 (13.4%) of the 632 malnutrition-related events and 56 (5.8%) of the 969 cases without malnutrition deemed causal or significant (table 2). Malnutrition was prevalent among children with known HIV-infection, even among those receiving antiretroviral therapy (ART), with the highest proportions in Mozambique (71.4%, 25/35) and Mali (77.8%, 7/9) (online supplemental eTable 4). Among 85 HIV-infected deaths with malnutrition, 35 (41.2%) had documented HIV testing, of which 32 (91.4%) were positive.

Among the 632 malnutrition-related deaths, 90.1% were underweight, 61.2% were stunted and 94.1% had wasting according to postmortem measurements (table 3, online supplemental eTable 3 and eFigure 3). Three hundred forty-five (54.6%) had all three conditions—underweight, stunting and wasting (online supplemental eTable 4). Among 547 decedents with malnutrition but without underlying HIV, malnutrition was classified as a mix of marasmus, kwashiorkor and other forms of protein-calorie malnutrition (table 1).

**Table 3:** Post-mortem anthropometric characteristics of infant and child deaths with malnutrition in causal chain or as other significant condition, CHAMPS, 2016–2023

Characteristic	Overall	1–5 months	6–11 months	12–23 months	24–59 months
	N=632	N=148	N=159	N=198	N=127
Weight-for-age Z-score (N=626)*					
Median (IQR)	−4.1 (−5.2, −3.0)	−4.7 (−5.9, –3.7)	−3.9 (−5.0, –2.8)	−4.1 (−4.8, –3.1)	−3.8 (−5.2, –2.3)
Mean (SD)	−4.1 (1.8)	−4.7 (1.6)	−4.0 (1.7)	−3.9 (1.9)	−3.8 (1.8)
Normal (≥ −2) (%)	62 (9.9)	5 (3.4)	16 (10.1)	19 (9.7)	22 (17.5)
Moderate underweight (−3 ≤ WAZ < −2) (%)	98 (15.7)	16 (11.0)	28 (17.6)	29 (14.9)	25 (19.8)
Severe underweight (< −3) (%)	466 (74.4)	125 (85.6)	115 (72.3)	147 (75.4)	79 (62.7)
Length-for-age Z-score (N=619)					
Median (IQR)	−2.6 (−4.1, –1.3)	−2.8 (−4.7, –1.6)	−1.9 (−3.2, –0.7)	−2.8(−3.9, –1.4)	−2.8 (−4.8, –1.5)
Mean (SD)	−2.8 (2.3)	−3.2 (2.4)	−2.2 (2.3)	−2.8 (2.1)	−3.1 (2.3)
Normal (≥ −2) (%)	240 (38.8)	47 (32.2)	80 (51.3)	68 (34.9)	45 (36.9)
Moderate stunting (−3 ≤ LAZ < −2) (%)	125 (20.2)	32 (21.9)	33 (21.2)	41 (21.0)	19 (15.6)
Severe stunting (< −3) (%)	254 (41.0)	67 (45.9)	43 (27.6)	86 (44.1)	58 (47.5)
Weight-for-length Z-score (N=605)					
Median (IQR)	−3.7 (−5.0, −2.4)	−3.7 (−5.2, –2.4)	−3.8 (−5.1, −2.5)	−3.7 (−5.1, –2.5)	−3.3 (−4.7, –1.9)
Mean (SD)	−3.6 (2.1)	−3.9 (2.3)	−3.8 (1.8)	−3.6 (2.2)	−3.1 (2.2)
Normal (≥ −2) (%)	109 (18.0)	25 (18.7)	17 (10.9)	36 (18.7)	31 (25.4)
Moderate wasted (−3 ≤ WLZ < −2) (%)	114 (18.8)	24 (17.9)	37 (23.7)	27 (14.0)	26 (21.3)
Severe wasted (< −3) (%)	382 (63.1)	85 (63.4)	102 (65.4)	130 (67.4)	65 (53.3)
Mid-upper arm circumference (cm) Z-score (N=551^a^)					
Median (IQR)	−3.4 (−4.8, –2.1)	−4.0 (−5.4, –3.0)	−3.4 (−4.6, –2.2)	−3.3 (−4.6, –2.1)	−3.4 (−4.8, –1.8)
Mean (SD)	−3.6 (2.0)	−4.1 (2.0)	−3.6 (2.0)	−3.4 (1.9)	−3.4 (2.1)
Normal (≥ −2) (%)	124 (22.5)	9 (12.5)	34 (21.7)	46 (23.5)	35 (27.8)
Moderate malnutrition (−3 ≤ MUACZ < −2) (%)	104 (18.9)	9 (12.5)	37 (23.6)	39 (19.9)	19 (15.1)
Severe malnutrition (< −3) (%)	323 (58.6)	54 (75.0)	86 (54.8)	111 (56.6)	72 (57.1)
Weight at MITS (kg) (N=629)					
Median (IQR)	5.7 (4.2, 7.3)	3.1 (2.2, 4.1)	5.5 (4.5, 6.2)	6.5 (5.6, 7.5)	8.5 (7.1, 10.4)
Mean (SD)	6.0 (2.6)	3.2 (1.2)	5.3 (1.3)	6.7 (2.1)	8.7 (2.4)
Any moderate to severe malnutrition (WAZ<-2 or LAZ<-2 or WLZ<-2 or MUACZ<-2) (%)	615 (97.5)	145 (98.0)	156 (98.1)	190 (96.0)	124 (98.4)
Any severe malnutrition (WAZ<-3 or LAZ<-3 or WLZ<-3 or MUACZ<-3) (%)	537 (85.1)	136 (91.9)	132 (83.0)	169 (85.4)	100 (79.4)

* Implausible anthropometric values for weight-for-age (WAZ<-10 or >5) (n=1), length-for-age (LAZ<-10 or >6) (n=10), weight-for-length (WLZ <-10 or >5) (n=6) and mid-upper arm circumference (MUACZ <-10 SD or >5) (n=3) were excluded.

LAZ, length-for-age Z-score; MITS, minimally invasive tissue sampling; MUACZ, mid-upper arm circumference Z-score; WAZ, weight-for-age Z-score; WLZ, weight-for-length Z-score.

**Figure 1:**
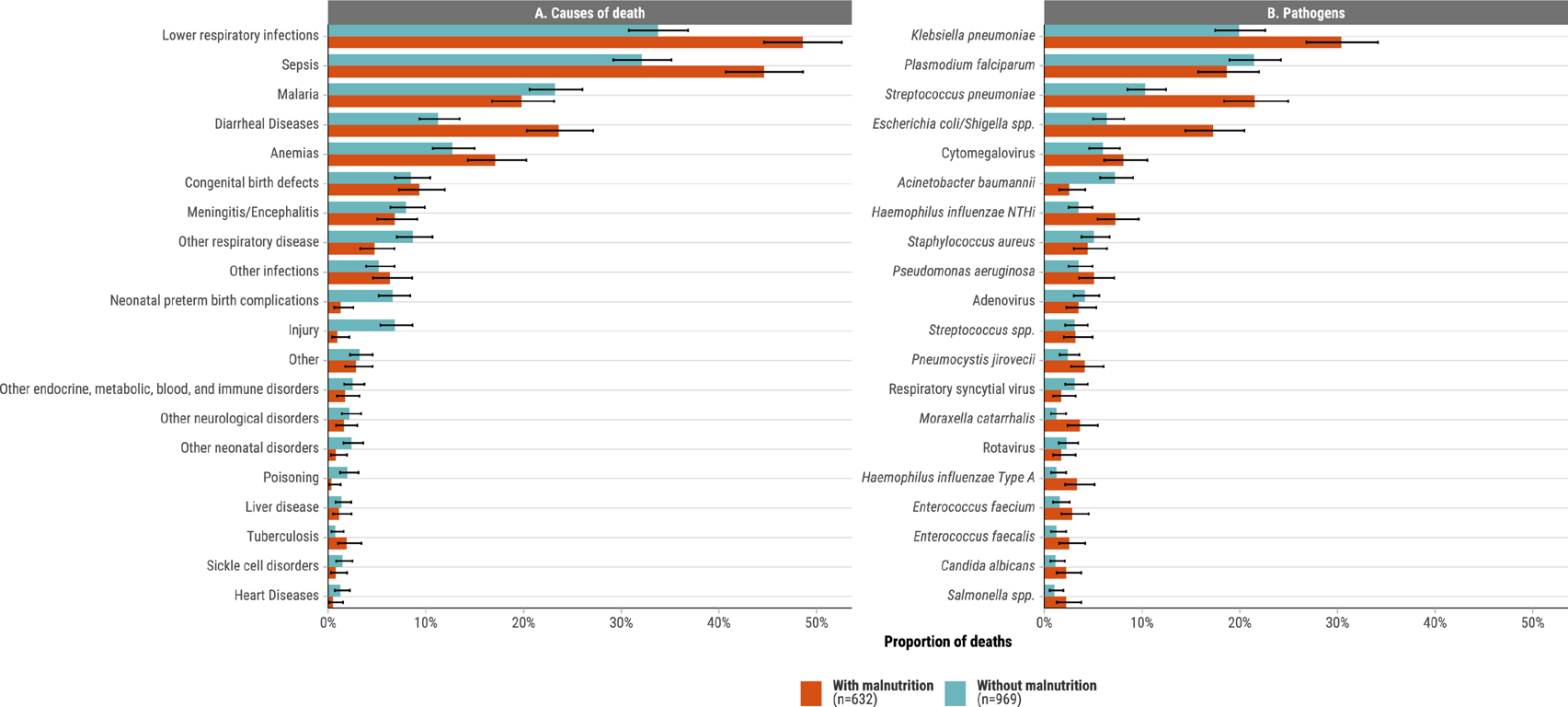
Causes of death (**A**) and pathogens in the causal pathway (**B**) for infant and child deaths with and without malnutrition in causal chain or as other significant condition. CHAMPS, 2016–2023 (N=1601). CHAMPS, Child Health and Mortality Prevention Surveillance.

The most frequent conditions in the causal chain of the 632 children with malnutrition-related deaths were lower respiratory infections (48.6%, n=307), sepsis (44.6%, n=282), diarrheal diseases (23.6%, n=149), malaria (19.8%, n=125) and anaemia ((17.1%, n=108), figure 1A, online supplemental eFigure 5), primarily serving as the immediate or antecedent causes when malnutrition was the underlying cause (online supplemental eFigure 6). Additionally, congenital defects were present in 59 (9.3%) malnutrition-related deaths, and neurological conditions in 10 (1.6%), almost all serving as the underlying cause when malnutrition was the immediate or antecedent cause (online supplemental eFigure 6). Congenital defects included cerebral palsy (n=12), Down syndrome (n=7) and congenital heart malformations (n=6). A greater proportion of malnutrition-related deaths (89.1%, 563/632) had one or more putative infectious diseases in the causal chain than malnutrition-unrelated deaths (77.3%, 749/969) (p<0.001). Ethiopia had a significantly higher proportion of malnutrition-related deaths with lower respiratory infections (84.6%, 66/78), sepsis (73.1%, 57/78) and meningitis (24.4%, 19/78) compared with malnutrition-related deaths from other sites combined (p<0.001) (online supplemental eTable 5). Among all 1601 decedents including those without malnutrition as a cause or significant condition, WAZ, LAZ, WLZ and MUACZ were significantly lower for deaths determined to have an infectious disease in the causal chain than deaths from other causes (p<0.001) (online supplemental eFigure 7). Among all 1601 decedents (including those without malnutrition), median WAZ (−2.95 vs −1.58), WHZ (−2.41 vs −1.76), HAZ (−1.82 vs −0.67) and MUACZ (−1.91 vs −0.88) scores were significantly lower for deaths with an infectious disease in the causal chain compared with deaths from other causes (p<0.001).

**Figure 2:**
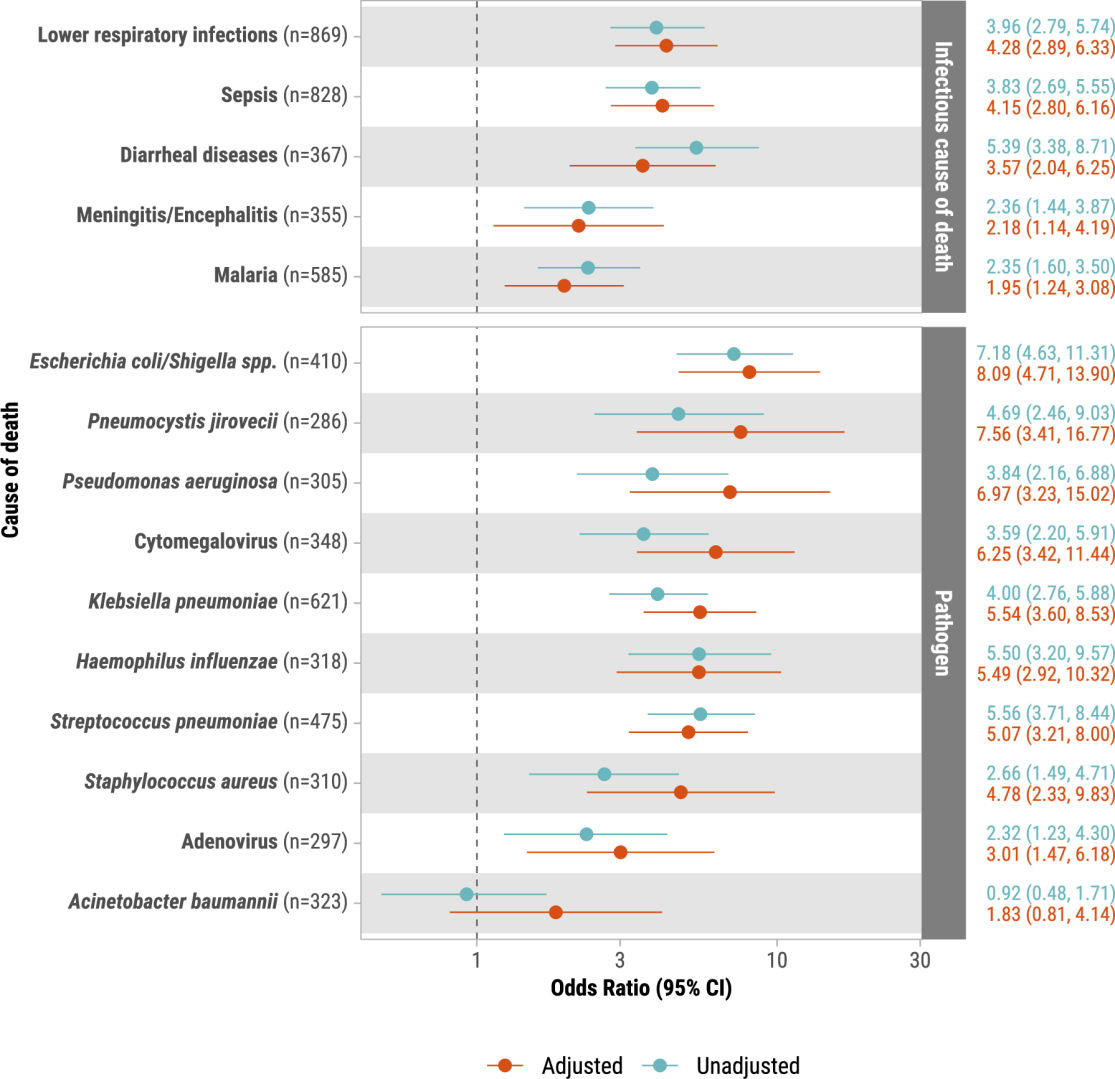
Unadjusted and adjusted associations between malnutrition in causal chain or as other significant condition and infectious causes of death in the causal chain among infant and child deaths. CHAMPS, 2016–2023. The x-axis is shown on a log_10_ scale. ORs and 95% CIs are shown. Multivariable models were adjusted for age group, sex, location of death and site as a random effect. The association between each cause of death and malnutrition was from separate regression models. This analysis excluded deaths from infectious causes from the reference groups. The sample size for each analysis is shown. CHAMPS, Child Health and Mortality Prevention Surveillance.

Adjusting for age group, sex, site and location of death (community vs healthcare facility), malnutrition-related deaths had higher odds of any infectious disease in the causal chain (aOR: 2.36, 95% CI 1.74 to 3.55) compared with deaths not attributed to malnutrition (online supplemental eFigure 8). Compared with deaths from non-infectious causes, the odds of having malnutrition were higher specifically for deaths from lower respiratory infections (aOR: 4.28, 95% CI 2.89 to 6.33), sepsis (aOR: 4.15, 95% CI 2.80 to 6.16), diarrheal diseases (aOR: 3.57, 95% CI 2.04 to 6.25) and malaria (aOR: 1.95, 95% CI 1.24 to 3.08) (figure 2A). Deaths that were underweight by measurements (WAZ <−2) had higher odds of any infectious disease in the causal chain (aOR: 2.04, 95% CI 1.41 to 2.95), as did those with stunting (HAZ <−2) (aOR: 1.56, 95% CI 1.11 to 2.19), compared with deaths having normal anthropometric weight and height, respectively (online supplemental eTable 6). Deaths that had all three anthropometric measure deficits (underweight, stunted and wasting) also had higher odds of any infectious disease in the causal chain (aOR: 3.51, 95% CI 2.33 to 5.27) compared with deaths within normal anthropometric range (online supplemental eTable 7).

Frequent pathogens in the causal pathway for 632 malnutrition-related deaths were *Klebsiella pneumoniae* (30.4%)*, Streptococcus pneumoniae* (21.5%)*, Plasmodium falciparum* (18.7%), *E. coli/Shigella spp* (17.2%), cytomegalovirus (8.1%) and non-typable *Haemophilus influenzae* (NTHi; 7.3%) (figure 1B, online supplemental eFigure 5B)). Adjusting for age group, sex, site and location of death, a higher odds of malnutrition as causal or significant condition were observed for deaths from *E. coli/Shigella* (aOR: 8.09, 95% CI 4.71 to 13.90), *Pneumocystis jirovecii* (aOR: 7.56, 95% CI 3.41 to 16.77), *Pseudomonas aeruginosa* (aOR: 6.97, 95% CI 3.23 to 15.02), cytomegalovirus (aOR: 6.25, 95% CI 3.42 to 11.44), *K. pneumoniae* (aOR: 5.54, 95% CI: 3.60 to 8.53), *H. influenzae* NTHi (aOR: 5.49, 95% CI 2.92 to 10.32), *S. pneumoniae* (aOR: 5.07, 95% CI 3.21 to 8.00), *Staphylococcus aureus* (aOR: 4.78, 95% CI 2.33 to 9.83) and adenovirus (aOR: 3.01, 95% CI 1.47 to 6.18) compared with deaths from non-infectious causes (figure 2B). Of the 109 malnutrition-related deaths with *E. coli/Shigella*, 90 (83.3%) tested positive on rectal swabs, 63 (57.8%) in blood and 19 (17.4%) in CSF (online supplemental eTable 8). Common coinfections among malnutrition-related cases were *K. pneumoniae* with *S. pneumoniae* (n=51) and *E. coli/Shigella* (n=49) and *S. pneumoniae* with *E. coli* (n=35) and non-typable *H. influenzae* (n=32) (online supplemental eFigure 9).

Most of the 632 malnutrition-related deaths (92.0%) were deemed preventable by the DeCoDe panel, significantly higher than the 80.5% considered preventable among 969 malnutrition-unrelated deaths (p<0.001) (table 2). Prevention recommendations included improved clinical management and quality of care (63.5%), health education (53.4%), nutritional support (52.7%) and promoting health-seeking behaviour (49.2%) (online supplemental eFigure 10).

## Discussion

Our detailed postmortem investigation from seven LMIC countries found malnutrition to be a major causal (30.8%) or other significant (8.8%) condition among infants and children, together implicated in 39.5% of deaths, most often as the underlying condition that contributed to a fatal outcome. Our estimates approximate those reported by WHO (45% of under-5 mortality) and provide additional validation of the large impact of malnutrition on child survival.^8^ Since anthropometric evidence of moderate-to-severe malnutrition was found in the majority of cases in our study (74%), including 36% of those deemed unrelated to malnutrition by the panel, these values are likely to be underestimated. Conceivably, the contribution of malnutrition may have been obscured by factors such as an incomplete clinical history or instances where the terminal event could have been fatal in the absence of malnutrition.

Most malnutrition met anthropometric criteria for severe, including 85% of those with malnutrition-related and 38% of unrelated, deaths. The occurrence of stunting in 61% of malnutrition-related deaths suggests that many children had longstanding nutritional faltering. However, stunting can also be influenced by other factors, such as underlying health conditions or intrauterine exposures that may have impeded growth. Considering evidence that the presence of multiple nutritional deficits forbodes a worse outcome,^23 5^,^7^ it is notable that most children in our study had more than one anthropometric measure indicating a moderate-to-severe deficit, and 54.6% had all three.

Our study highlights the vicious cycle of malnutrition and infectious diseases that is well described.^24^,^27^ Infectious diseases such as diarrhoea can exacerbate malnutrition via anorexia, intestinal injury, malabsorption and enhanced urinary nitrogen loss.^28 29^ In turn, malnutrition predisposes to immune dysfunction and increased susceptibility to infection. Accordingly, among malnutrition-related deaths in CHAMPS, 89.1% had infectious diseases in the causal chain, significantly higher than cases without malnutrition. Lower respiratory infections, sepsis, malaria and diarrheal diseases were seen most often, as reported elsewhere.^30^,^33^ Common pathogens were *K. pneumoniae, S. pneumoniae,* enteropathogenic *E. coli* and cytomegalovirus. Further understanding the mechanisms of malnutrition and infectious morbidity can inform targeted interventions and treatments to address nutritional deficiencies and prevent malnutrition-associated deaths.

Over half of deaths among HIV-infected children had HIV-related wasting syndrome, in line with other studies.^34^ A meta-analysis of HIV-positive children in East Africa reported pooled prevalences across 22 studies of approximately 42% for underweight, 25% for wasting and 50% for stunting.^34^ Factors contributing to nutritional deficits in HIV-infected children include anorexia, catabolism, HIV-induced enteropathy, which can lead to malabsorption of food and medications and frequent infections.^1435^,^37^ In turn, malnutrition has been shown to interact with HIV infection to accelerate morbidity and mortality.^35 36^ Early onset of diarrhoea (<6 months old) in HIV-infected infants has been associated with the later development of persistent diarrhoea, and those with persistent episodes had more severe HIV infection, characterised by a significantly higher frequency of opportunistic infections and lower CD4+ T-lymphocyte counts by 1 year of age.^37^ Although HIV-related wasting is well-known, malnutrition challenges persist even among children on ART in resource-limited settings.^14^ Significant variability in the prevalence of malnutrition among HIV-infected children necessitates further research into contributing factors and improved interventions, particularly in high-burden regions. In addition, WHO guidelines recommend that children with SAM HIV-endemic areas should be routinely tested for the virus.^38^

Our study’s findings align with established recommendations for preventing malnutrition-related deaths, highlighting the need for broader implementation and addressing common challenges.^39^,^44^ As previously suggested by the WHO, integrated interventions promoting optimal infant and young child feeding practices, timely micronutrient supplementation, effective management of childhood illness and improved maternal health remain crucial strategies.^45^ A previous CHAMPS analysis revealed gaps in service delivery, with only 14% of children who died from malnutrition receiving treatment.^46 47^ Strengthening healthcare systems and addressing resource limitations, particularly ready-to-use therapeutic foods and trained healthcare staff, are critical for improving outcomes.^48^

This study is subject to several limitations. Due to the inherent subjectivity in diagnosing both malnutrition and infectious disease as CoD, particularly when considered together by DeCoDe panels, the observed association between these factors may be inflated by diagnostic bias (eg, Berkson’s bias). The study design, analysing cause of death data (conditioning on death), may introduce collider bias, potentially underestimating the true association between infectious diseases and malnutrition. The increased specificity of diagnoses determined through the comprehensive, standardised data collection and the DeCoDe adjudication process in conjunction with anthropometric measurements using quality-controlled instruments and trained personnel obtained at the time of each MITS procedure allows a more precise diagnosis of malnutrition and estimation of cause-specific prevalence of malnutrition. Nonetheless, deciphering the temporal sequence of malnutrition and infections posed challenges. Historical information on the conditions that predisposed to malnutrition is often lacking. Despite standardisation of processes and cross-site quality assurance, there may be site-to-site variation in the proportion of deaths deemed related to malnutrition. Although CHAMPS has been highly successful in enrolling cases across sites, acceptability of MITS poses limitations to the ability to enrol a representative sample of community and facility-based deaths.^15 16^ Postmortem swelling, often resulting from rapid decomposition and gas discharge, can introduce challenges to precise measurements. The cross-sectional nature of the CHAMPS data limit our ability to definitively establish a causal relationship between malnutrition and mortality. Although we can identify a high prevalence of malnutrition in deceased children, a longitudinal study design would be necessary to definitively assess the temporal sequence and causal contribution of malnutrition to these deaths. The absence of conventional control groups hinders direct contrasts between deceased and living children, as CHAMPS data show the contribution of malnutrition in cases where medical intervention fell short. The limited number of infant and child deaths without infectious diseases constrained the scope of regression analyses, leading to wider 95% CIs. Nonetheless, while CoD assignment involves clinical judgement by DeCoDe panel members, the extensive data available enables more accurate CoD attribution than methods relying solely on measurements or limited clinical diagnostics.

## Conclusion

Malnutrition was a causal or significant factor in 4 out of 10 under-5 deaths in the CHAMPS network, often occurring alongside infectious diseases. These findings emphasise the critical need for integrated interventions that address both malnutrition and infectious diseases to effectively reduce child mortality. Strengthening systems for early detection and treatment of malnutrition, particularly in resource-limited settings, could substantially reduce under-5 mortality. Achieving sustainable progress will require approaches that account for the social determinants underlying malnutrition, such as poverty, food insecurity, climate change, conflict, gender roles, and inadequate access to healthcare.

## Supplementary material

10.1136/bmjgh-2024-017262 online supplemental file 1

## Data Availability

Data are available upon reasonable request.
