# Supplementary material for: Contribution of malnutrition to infant and child deaths in Sub-Saharan Africa and South Asia
Source: BMJ Glob Health. 2024 Dec 5;9(12):e017262. doi: 10.1136/bmjgh-2024-017262 (PMC11624724; doi:10.1136/bmjgh-2024-017262)
Supplement: online supplemental file 1 [file bmjgh-9-12-s001.pdf]

Supplemental Online Content

eAppendix 1. Supplemental methods.

eTable 1. Anthropometric characteristics of infant and child deaths with and without malnutrition as causal or significant condition by age group, CHAMPS, 2016–2023 (N=1601).

eTable 2. Comparison of characteristics between infant and child deaths with malnutrition as causal or significant condition, CHAMPS, 2016–2023 (N=632)

eTable 3. Anthropometric characteristics and causal chain classifications of infant and child deaths with malnutrition as causal or significant condition by site, CHAMPS, 2016–2023 (N=632).

eTable 4. Proportion of deaths with HIV infection that had malnutrition as causal or significant condition and proportion that had clinical record HIV testing and of receiving antiretroviral therapy for treatment by site, CHAMPS, 2016–2023 (N=141).

eTable 5. Other causes of death in the causal chain for infant and child deaths with malnutrition as causal or significant condition by site, CHAMPS, 2016–2023 (N=632).

eTable 6. Associations between individual measures of postmortem anthropometric malnutrition and infectious causes in the causal chain, CHAMPS, 2016–2023 (N = 1526).

eTable 7. Associations between combinations of postmortem anthropometric malnutrition measures and infectious causes in the causal chain, CHAMPS, 2016–2023 (N = 1526).

eTable 8. Positive TAC PCR tests by specimen for 109 deaths with malnutrition as causal or significant condition attributed to *Escherichia coli/Shigella*.

eFigure 1. Flowchart of enrolled under-five infant and child deaths from CHAMPS sites between December 2016 – December 2023, that had minimally invasive tissue samples (MITS) and consent only for verbal autopsy and clinical abstraction (non-MITS) and included in the analysis.

eFigure 2. Flowchart of enrolled under-five infant and child deaths from CHAMPS sites between December 2016 – December 2023, that had minimally invasive tissue samples (MITS) and consent only for verbal autopsy and clinical abstraction (non-MITS) and included in the analysis by site.

eFigure 3. Anthropometric measures for infant and child deaths with malnutrition as causal or significant condition, CHAMPS, 2016–2023 (N=632).

eFigure 4. Venn diagram of the proportion of deaths with malnutrition as causal or significant condition who were wasted (weight-for-height z-score < -2 or mid-upper arm circumference z-score < -2), stunted (height-for-age z-score < -2), or underweight (weight-for-age z-score < -2), CHAMPS, 2016–2023 (N=632).

eFigure 5. Other causes of death (A) and pathogens (B) when malnutrition is a causal or significant condition, CHAMPS, 2016–2023 (N=632).

eFigure 6. Underlying causes of death (A) and pathogens (B) when malnutrition is the antecedent/immediate cause of death (red), and antecedent/immediate causes of death and pathogens when malnutrition is the underlying cause of death (blue), CHAMPS, 2016–2023 (N=493).

eFigure 7. Boxplots of anthropometric measures for infant and child deaths with infectious and non-infectious causes of death, CHAMPS, 2016–2023 (N=1601).

eFigure 8. Unadjusted and adjusted odds of a death having malnutrition as causal or significant condition for the most frequent causes of death in the causal chain among infant and child deaths, CHAMPS, 2016–2023 (N = 1601).

eFigure 9. Frequencies of co-infections among infant and child deaths with malnutrition as causal or other significant condition, CHAMPS, 2016–2023 (N = 632).

eFigure 10. Expert (DeCoDe) panel recommendations for preventing deaths with malnutrition as causal or other significant condition, CHAMPS, 2016–2023 (N = 526).

References.

eAppendix 1. Supplemental methods.

Data Sources

The CHAMPS database contains comprehensive information on all stillbirths and <5 deaths enrolled at each surveillance site. These data include demographics, postmortem diagnostic results, verbal autopsy data, clinical chart abstraction data for each child and, when available, maternal antenatal records. Deaths identified within 24 hours (or 72 hours if the body was refrigerated) were eligible for enrollment. Detailed information regarding site characteristics, selection criteria, catchment areas, death notification methods, eligibility screening, and specimen and data collection methods have been previously described.[1,2]

Details regarding the cause of death determination and standardization processes across sites can be found elsewhere.[2,3] Briefly, if caregivers consent, deaths are investigated using minimally invasive tissue sampling (MITS), a postmortem approach using biopsy needles for sampling key organs and body fluids. These samples are subjected to testing using conventional microbiology and multiplexed polymerase chain reaction (PCR) assays using TaqMan array cards. Additionally, pathologists conduct examinations of the tissues and perform advanced histopathological tests. Information concerning the terminal events leading to death is abstracted from medical records and through family interviews using the standard verbal autopsy tool.

At each surveillance site, a determination of cause of death (DeCoDe) panel consisting of pediatricians, obstetricians, epidemiologists, pathologists, microbiologists, and other healthcare providers review case data to assign causes of death. WHO International Classification of Diseases 10 (ICD-10) and WHO application of ICD-10 deaths during the perinatal period (ICD-PM) are used by CHAMPS for coding purposes.[4,5] For deaths in which only a single cause led to death, that cause is listed as the underlying cause. For deaths in which multiple causes led to the death, the panel determines the causal chain including the underlying, antecedent, and immediate causes leading to death.[6] The underlying cause usually occurred before immediate or antecedent conditions and may have predisposed the child to an immediate or antecedent illness that then led to death; the immediate cause was closest to the death and the antecedent causes were in-between the underlying and immediate causes. Each death has only one underlying cause, zero or one immediate cause, and zero to multiple antecedent causes. The panel also reports other conditions that, while not

directly causing death, hold significance in the overall context. As a quality control measure, a subset of cases that underwent DeCoDe review at the site level are shared with other sites for secondary review. The DeCoDe panel determines whether each death was preventable by considering all available information, including demographic, clinical, pathological, microbiological, verbal autopsy, photography, and anthropometric measurements. If the death is deemed potentially preventable, the panel identifies specific health system gaps and recommends improvements that could have averted the death.[7]

Statistical analysis

We reported medians and interquartile ranges [IQR] for continuous variables (age, hospital duration, time from death to MITS) and frequency distributions for categorical variables (age group, sex, location of death, site, HIV status, antemortem diagnosis, count of causal conditions identified, birthweight, preventable) for infant and child deaths with and without malnutrition as causal or significant conditions.

For deaths with malnutrition as causal or significant condition, we reported medians with IQRs and means with standard deviations for anthropometric characteristics (WAZ, HAZ, WHZ, MUACZ, weight at MITS) stratified by age group (1–5 months, 6–11 months, 12–23 months, 24–59 months) and site. Furthermore, we evaluated anthropometric characteristics for deaths with and without malnutrition as causal or significant condition by age group. We reported specific ICD-10 codes associated with malnutrition and frequencies of deaths with malnutrition classified as underlying, antecedent, or immediate causes of death, or as other significant conditions.

Next, we examined other causes of death and pathogens in the causal pathway for deaths with malnutrition as causal or significant conditions and deaths resulting from other causes. We also reported underlying causes of death and pathogens when malnutrition was the antecedent or immediate cause of death, and antecedent or immediate causes of death and pathogens when malnutrition was the underlying cause of death. Moreover, we investigated frequencies of co-infections among deaths with malnutrition in the causal chain and used Spearman's coefficients to assess correlations between pairs of pathogens, considering significance at $p<0.05$.

We used mixed-effect logistic regression to evaluate associations between the presence or absence of malnutrition as causal or significant conditions and:

1) Other causes of death in the causal chain among all infant and child deaths. The association between malnutrition and each cause of death (sepsis, diarrheal diseases, lower respiratory infections, anemias, congenital birth defects, other infections, meningitis/encephalitis, malaria, other respiratory disease) was evaluated in separate regression models. For example, the association between malnutrition and sepsis compared deaths from sepsis to deaths from all causes other than sepsis. While focusing on the most prevalent causes of death, we also evaluated associations between malnutrition and any infectious disease in the causal chain (congenital infection, lower respiratory infections, diarrheal diseases with an identified etiologic agent, malaria, measles, meningitis/encephalitis, other infections, rabies, sepsis, syphilis, tuberculosis, upper respiratory infections). See eFigure 8.
2) Infectious causes of death (sepsis, diarrheal diseases, lower respiratory infections, meningitis/encephalitis, malaria), excluding deaths from infectious causes from the reference groups. For example, rather than comparing sepsis-attributed deaths to all non-sepsis deaths, this sub-analysis excluded deaths from lower respiratory infections, malaria, and all other infectious diseases from the reference group. See Figure 2.
3) Pathogens in the causal chain. The association between malnutrition and each pathogen (*Escherichia coli, Pseudomonas aeruginosa, Klebsiella pneumoniae, Streptococcus pneumoniae, Haemophilus influenzae, Staphylococcus aureus, Pneumocystis jirovecii, Acinetobacter baumannii, Plasmodium falciparum,* cytomegalovirus, adenovirus) was evaluated in separate regression models. This analysis also excluded deaths from infectious causes from the reference groups. See Figure 2.

Multivariable analyses for 1), 2), and 3) were adjusted for age group, sex, and location of death, with site included as a random effect.

We also used mixed-effect logistic regression to evaluate associations between the presence of postmortem anthropometric malnutrition, and any infectious disease in the causal chain. This analysis defined stunting (LAZ<-2), wasting (WHZ<-2 or MUACZ<-2), and underweight (WAZ<-2) by postmortem anthropometric measures which may include some deaths without malnutrition as causal or significant conditions. Specifically, we:

4) Examined associations between individual anthropometric indicators (stunting, wasting, and underweight) and the presence of any infectious disease. Stunting, wasting, and underweight were included in the same model as separate independent variables with reference groups no stunting, no wasting, and not underweight, respectively. See eTable 6.

5) Constructed a variable representing different combinations of anthropometric indicators using dummy codes and analyzed its association with infectious diseases. The reference group for this analysis included deaths with normal anthropometric measures (LAZ≥-2 & WLZ≥-2 & WAZ≥-2 & MUACZ≥-2). See eTable 7.

4) and 5) were complete case analyses excluding deaths missing anthropometric values or with implausible anthropometric measures for stunting, wasting, or underweight. HIV as a chronic disease could predispose a child to both infection and malnutrition, therefore 4) and 5) also include HIV infection in the multivariable models. We evaluated interaction terms between HIV and stunting, HIV and wasting, and HIV and underweight. Odds ratios and 95% confidence intervals were reported, and statistical significance was set at $p<0.05$. We used Hosmer-Lemeshow C and H statistics to evaluate overall goodness-of-fit for the regression models. Tolerance values were used to examine potentially collinearity in the multivariable models.

Finally, we evaluated the proportion of deaths with malnutrition as causal or significant conditions that were deemed preventable. For deaths that were considered preventable, we reported the recommendations provided by the expert DeCoDe panel on how such deaths could have been averted.

eTable 1. Anthropometric characteristics of infant and child deaths with and without malnutrition as causal or significant condition by age group, CHAMPS, 2016–2023 (N=1601).

Characteristic	Overall		1-5 months		6-11 months		12-23 months		24-59 months	
	With malnutrition N = 632 Median [IQR]	Without malnutrition N = 969 Median [IQR]	With malnutrition N = 148 Median [IQR]	Without malnutrition N = 370 Median [IQR]	With malnutrition N = 159 Median [IQR]	Without malnutrition N = 160 Median [IQR]	With malnutrition N = 198 Median [IQR]	Without malnutrition N = 182 Median [IQR]	With malnutrition N = 127 Median [IQR]	Without malnutrition N = 257 Median [IQR]
**Weight-for-age Z-score** (N=1565)										
Median [IQR]	-4.1 [-5.2, -3.0]	-1.6 [-3.1, -0.6]	-4.7 [-5.9, -3.7]	-2.6 [-5.0, -1.2]	-3.9 [-5.0, -2.8]	-1.1 [-2.0, 0.0]	-4.1 [-4.8, -3.1]	-1.3 [-2.1, -0.3]	-3.8 [-5.2, -2.3]	-1.3 [-2.1, -0.4]
Normal (≥ -2) (%)	62 (9.9)	552 (58.8)	5 (3.4)	135 (37.3)	16 (10.1)	116 (74.4)	19 (9.7)	126 (71.6)	22 (17.5)	175 (71.4)
Moderate underweight (-3 ≤ WAZ < -2) (%)	98 (15.7)	145 (15.4)	16 (11.0)	64 (17.7)	28 (17.6)	17 (10.9)	29 (14.9)	25 (14.2)	25 (19.8)	39 (15.9)
Severe underweight (< -3) (%)	466 (74.4)	242 (25.8)	125 (85.6)	163 (45.0)	115 (72.3)	23 (14.7)	147 (75.4)	25 (14.2)	79 (62.7)	31 (12.7)
**Length-for-age Z-score** (N=1552)										
Median [IQR]	-2.6 [-4.1, -1.3]	-0.8 [-2.5, 0.3]	-2.8 [-4.7, -1.6]	-1.6 [-4.1, 0.1]	-1.9 [-3.2, -0.7]	-0.2 [-1.5, 0.9]	-2.8 [-3.9, -1.4]	-0.6 [-1.8, 0.2]	-2.8 [-4.8, -1.5]	-0.9 [-2.1, 0.1]
Normal (≥ -2) (%)	240 (38.8)	652 (69.9)	47 (32.2)	200 (56.8)	80 (51.3)	128 (83.7)	68 (34.9)	139 (78.5)	45 (36.9)	185 (73.7)
Moderate stunting (-3 ≤ LAZ < -2) (%)	125 (20.2)	95 (10.2)	32 (21.9)	30 (8.5)	33 (21.2)	11 (7.2)	41 (21.0)	18 (10.2)	19 (15.6)	36 (14.3)
Severe stunting (< -3) (%)	254 (41.0)	186 (19.9)	67 (45.9)	122 (34.7)	43 (27.6)	14 (9.2)	86 (44.1)	20 (11.3)	58 (47.5)	30 (12.0)
**Weight-for-length Z-score** (N=1471)										
Median [IQR]	-3.7 [-5.0, -2.4]	-1.5 [-2.7, -0.3]	-3.7 [-5.2, -2.4]	-1.8 [-3.8, -0.7]	-3.8 [-5.1, -2.5]	-1.3 [-2.6, -0.2]	-3.7 [-5.1, -2.5]	-1.3 [-2.5, 0.0]	-3.3 [-4.7, -1.9]	-1.2 [-2.0, -0.2]
Normal (≥ -2) (%)	109 (18.0)	556 (64.2)	25 (18.7)	158 (51.1)	17 (10.9)	102 (67.5)	36 (18.7)	118 (68.2)	31 (25.4)	178 (76.4)
Moderate wasted (-3 ≤ WLZ < -2) (%)	114 (18.8)	123 (14.2)	24 (17.9)	47 (15.2)	37 (23.7)	19 (12.6)	27 (14.0)	25 (14.5)	26 (21.3)	32 (13.7)
Severe wasted (< -3) (%)	382 (63.1)	187 (21.6)	85 (63.4)	104 (33.7)	102 (65.4)	30 (19.9)	130 (67.4)	30 (17.3)	65 (53.3)	23 (9.9)
**Mid-upper arm circumference (cm) Z-score** (N=1296)										
Median [IQR]	-3.4 [-4.8, -2.1]	-0.7 [-1.7, 0.3]	-4.0 [-5.4, -3.0]	-1.3 [-3.2, -0.3]	-3.4 [-4.6, -2.2]	-0.5 [-1.6, 0.4]	-3.3 [-4.6, -2.1]	-0.5 [-1.5, 0.5]	-3.4 [-4.8, -1.8]	-0.5 [-1.3, 0.3]
Normal (≥ -2) (%)	124 (22.5)	598 (80.3)	9 (12.5)	93 (61.6)	34 (21.7)	132 (83.0)	46 (23.5)	150 (82.9)	35 (27.8)	223 (87.8)
Moderate malnutrition (-3 ≤ MUACZ < -2) (%)	104 (18.9)	65 (8.7)	9 (12.5)	17 (11.3)	37 (23.6)	10 (6.3)	39 (19.9)	16 (8.8)	19 (15.1)	22 (8.7)
Severe malnutrition (< -3) (%)	323 (58.6)	82 (11.0)	54 (75.0)	41 (27.2)	86 (54.8)	17 (10.7)	111 (56.6)	15 (8.3)	72 (57.1)	9 (3.5)
**Weight at MITS (kg)** (N=1583)	5.7 [4.2, 7.3]	7.6 [4.6, 10.5]	3.1 [2.2, 4.1]	4.0 [2.8, 5.4]	5.5 [4.5, 6.2]	7.5 [6.6, 8.5]	6.5 [5.6, 7.5]	9.0 [8.2, 10.0]	8.5 [7.1, 10.4]	12.6 [10.6, 14.5]
**Any moderate to severe malnutrition** (LAZ<-2 or WLZ<-2 or WAZ<-2 or MUACZ<-2) (%)	615 (97.5)	573 (59.2)	145 (98.0)	287 (77.6)	156 (98.1)	71 (44.4)	190 (96.0)	91 (50.0)	124 (98.4)	124 (48.4)
**Any severe malnutrition** (LAZ<-3 or WLZ<-3 or WAZ<-3 or MUACZ<-3) (%)	537 (85.1)	371 (38.3)	136 (91.9)	227 (61.4)	132 (83.0)	43 (26.9)	169 (85.4)	46 (25.3)	100 (79.4)	55 (21.5)

IQR: interquartile range; SD: standard deviation; MUACZ: mid-upper arm circumference Z-score; LAZ: length-for-age Z-score; WAZ: weight-for-age Z-score; WLZ: weight-for-length Z-score; MITS: minimally invasive tissue sampling

eTable 2. Comparison of characteristics between infant and child deaths with malnutrition as causal or significant condition, CHAMPS, 2016–2023 (N=632).

	Malnutrition: causal chain N=493[a]	Malnutrition: other significant condition N=139	*p*-value
**Age in days** (median [IQR])	380 [213, 640]	353 [164, 724]	0.646
**Age group** (%)			0.033
1-5 months	107 (21.7)	41 (29.5)	
6-11 months	127 (25.8)	32 (23.0)	
12-23 months	166 (33.7)	32 (23.0)	
24-59 months	93 (18.9)	34 (24.5)	
**Male sex** (%)	268 (54.4)	73 (52.5)	0.773
**Location of death** (%)			0.410
Community	166 (33.7)	41 (29.5)	
Facility	327 (66.3)	98 (70.5)	
**Hospital duration in hours** (median [IQR]) (N=362)	38 [11, 127]	57 [17, 211]	0.052
**Site** (%)			<0.001
Bangladesh	4 (0.8)	0 (0.0)	
Ethiopia	70 (14.2)	8 (5.8)	
Kenya	136 (27.6)	28 (20.1)	
Mali	51 (10.3)	7 (5.0)	
Mozambique	63 (12.8)	42 (30.2)	
Sierra Leone	138 (28.0)	19 (13.7)	
South Africa	31 (6.3)	35 (25.2)	
**Time from death to MITS in hours** (median [IQR])	10 [4, 17]	14 [6, 19]	0.004
**Count of causal conditions identified** (%)			<0.001
0	0 (0.0)	5 (3.6)	
1	13 (2.6)	57 (41.0)	
2	124 (25.2)	44 (31.7)	
3	183 (37.1)	17 (12.2)	
≥4	173 (35.1)	16 (11.5)	
Median [IQR]	3 [2, 4]	2 [1, 2]	<0.001
**Deemed preventable from DeCoDe panel** (%)	455 (92.3)	112 (80.6)	<0.001
**Weight-for-age Z-score** (median [IQR]) (N=626)	-4.4 [-5.5, -3.3]	-3.2 [-4.3, -2.2]	<0.001
**Length-for-age Z-score** (median [IQR]) (N=619)	-2.7 [-4.3, -1.4]	-2.1 [-3.3, -0.8]	0.003
**Weight-for-length Z-score** (median [IQR]) (N=605)	-3.9 [-5.2, -2.7]	-2.5 [-3.9, -1.6]	<0.001
**Mid-upper arm circumference (cm) Z-score** (median [IQR]) (N=551)	-3.6 [-5.0, -2.3]	-2.8 [-3.9, -1.5]	<0.001
**Weight at MITS (kg)** (median [IQR]) (N=629)	5.6 [4.1, 7.0]	6.3 [4.5, 8.1]	0.003

IQR: interquartile range; MITS: minimally invasive tissue sampling; MITS: minimally invasive tissue sampling; DeCoDe: Determination of Cause of Death panel.

[a] Two deaths that had malnutrition listed in the causal chain and as another significant condition are shown in the causal chain column.

eTable 3. Anthropometric characteristics and causal chain classifications of infant and child deaths with malnutrition as causal or significant condition by site, CHAMPS, 2016–2023 (N=632).

Characteristic	Bangladesh N = 4	Ethiopia N = 78	Kenya N = 164	Mali N = 58	Mozambique N = 105	Sierra Leone N = 157	South Africa N = 66
**Weight-for-age Z-score** (N=626)							
Median [IQR]	-4.4 [-4.7, -3.6]	-5.0 [-6.0, -3.9]	-4.3 [-5.1, -3.2]	-3.8 [-5.5, -2.8]	-4.1 [-5.2, -3.0]	-3.8 [-4.7, -2.7]	-4.0 [-5.0, -2.7]
Mean (SD)	-4.1 (1.1)	-4.9 (1.7)	-4.2 (1.7)	-4.0 (1.8)	-4.2 (1.7)	-3.8 (1.6)	-3.8 (2.3)
Normal (≥-2) (%)	0 (0.0)	5 (6.4)	13 (7.9)	7 (12.5)	10 (9.5)	14 (8.9)	13 (20.6)
Moderate underweight (-3≤WAZ<-2) (%)	1 (33.3)	7 (9.0)	22 (13.4)	11 (19.6)	17 (16.2)	36 (22.9)	4 (6.3)
Severe underweight (<-3) (%)	2 (66.7)	66 (84.6)	129 (78.7)	38 (67.9)	78 (74.3)	107 (68.2)	46 (73.0)
**Length-for-age Z-score** (N=619)							
Median [IQR]	-2.2 [-2.8, -1.8]	-4.9 [-6.2, -3.7]	-2.3 [-3.6, -1.4]	-2.0 [-3.4, -1.1]	-3.3 [-4.7, -2.2]	-2.0 [-3.1, -0.8]	-2.2 [-3.3, -0.2]
Mean (SD)	-2.4 (1.2)	-4.9 (2.2)	-2.5 (1.8)	-2.3 (1.9)	-3.6 (2.4)	-2.1 (1.9)	-2.1 (2.7)
Normal (≥-2) (%)	2 (50.0)	8 (10.7)	69 (42.1)	28 (50.0)	23 (22.5)	79 (51.0)	31 (49.2)
Moderate stunting (-3≤HAZ<-2) (%)	1 (25.0)	7 (9.3)	43 (26.2)	8 (14.3)	21 (20.6)	31 (20.0)	14 (22.2)
Severe stunting (<-3) (%)	1 (25.0)	60 (80.0)	52 (31.7)	20 (35.7)	58 (56.9)	45 (29.0)	18 (28.6)
**Weight-for-length Z-score** (N=605)							
Median [IQR]	-3.3 [-4.0, -2.9]	-2.7 [-4.2, -1.7]	-4.2 [-5.3, -3.0]	-3.7 [-5.5, -2.9]	-3.0 [-4.4, -1.8]	-3.7 [-4.9, -2.8]	-3.9 [-6.0, -1.1]
Mean (SD)	-3.5 (1.1)	-2.9 (2.1)	-4.2 (1.8)	-4.0 (2.0)	-2.9 (2.2)	-3.7 (1.7)	-3.6 (3.2)
Normal (≥-2) (%)	0 (0.0)	24 (32.9)	17 (10.4)	4 (7.0)	30 (30.6)	13 (8.6)	21 (35.6)
Moderate wasted (-3≤WHZ<-2) (%)	1 (33.3)	16 (21.9)	24 (14.7)	11 (19.3)	19 (19.4)	40 (26.3)	3 (5.1)
Severe wasted (<-3) (%)	2 (66.7)	33 (45.2)	122 (74.8)	42 (73.7)	49 (50.0)	99 (65.1)	35 (59.3)
**Mid-upper arm circumference (cm) Z-score** (N=551)							
Median [IQR]	-4.3 [-5.9, -3.2]	-4.4 [-5.8, -3.2]	-3.6 [-5.0, -2.3]	-3.1 [-4.7, -2.1]	-3.8 [-5.2, -2.4]	-2.6 [-3.8, -1.7]	-3.1 [-4.5, -1.7]
Mean (SD)	-4.7 (2.7)	-4.6 (2.0)	-3.8 (1.8)	-3.4 (2.0)	-3.8 (2.1)	-2.9 (1.7)	-3.1 (2.2)
Normal (≥-2) (%)	0 (0.0)	6 (9.0)	29 (18.8)	9 (19.6)	20 (21.5)	47 (33.6)	13 (27.1)
Moderate malnutrition (-3≤MUACZ<-2) (%)	1 (33.3)	8 (11.9)	23 (14.9)	14 (30.4)	13 (14.0)	37 (26.4)	8 (16.7)
Severe malnutrition (<-3) (%)	2 (66.7)	53 (79.1)	102 (66.2)	23 (50.0)	60 (64.5)	56 (40.0)	27 (56.2)
**Weight at MITS (kg)** (N=629)							
Median [IQR]	4.6 [3.6, 6.9]	5.7 [3.7, 7.2]	5.6 [4.4, 6.8]	6.1 [4.0, 7.4]	6.0 [4.3, 8.0]	6.2 [4.9, 7.5]	4.7 [3.0, 6.3]
Mean (SD)	5.5 (3.4)	5.5 (2.4)	6.0 (2.5)	6.2 (2.7)	6.2 (2.6)	6.2 (2.5)	5.2 (3.1)
**Any moderate to severe malnutrition** (LAZ<-2 or WLZ<-2 or WAZ<-2 or MUACZ<-2) (%)	4 (100.0)	77 (98.7)	161 (98.2)	55 (96.5)	103 (98.1)	154 (98.1)	61 (92.4)
**Any severe malnutrition** (LAZ<-3 or WLZ <-3 or WAZ<-3 or MUACZ<-3) (%)	4 (100.0)	74 (94.9)	140 (85.4)	47 (82.5)	94 (89.5)	122 (77.7)	56 (84.8)
**Malnutrition classified as:**							
Underlying cause of death	3 (75.0)	65 (83.3)	111 (67.7)	20 (34.5)	47 (44.8)	108 (68.8)	22 (33.3)
Immediate cause of death	0 (0.0)	0 (0.0)	0 (0.0)	1 (1.7)	1 (1.0)	0 (0.0)	0 (0.0)
Antecedent cause of death	1 (25.0)	5 (6.4)	27 (16.5)	31 (53.4)	15 (14.3)	30 (19.1)	9 (13.6)
Other significant condition	0 (0.0)	8 (10.3)	29 (17.7)	7 (12.1)	43 (41.0)	19 (12.1)	35 (53.0)

IQR: interquartile range; SD: standard deviation; MUACZ: mid-upper arm circumference Z-score; LAZ: height-for-age Z-score; WAZ: weight-for-age Z-score; WLZ: weight-for-height Z-score; MITS: minimally invasive tissue sampling

eTable 4. Proportion of deaths with HIV infection that had malnutrition as causal or significant condition and proportion that had clinical record of HIV testing and of receiving antiretroviral therapy for treatment by site, CHAMPS, 2016–2023 (N=141).

Site	Total HIV-infected deaths N = 141 n/N (%)	Ethiopia N = 1 n/N (%)	Kenya N = 36 n/N (%)	Mali N = 9 n/N (%)	Mozambique N = 35 n/N (%)	Sierra Leone N = 27 n/N (%)	South Africa N = 33 n/N (%)
**HIV infected with malnutrition**	**85/141 (60.3)**	**0/1 (0.0)**	**23/36 (63.9)**	**7/9 (77.8)**	**25/35 (71.4)**	**15/27 (55.6)**	**15/33 (45.5)**
Any positive antemortem HIV test[a]	32/35 (91.4)	–	5/6 (83.3)	–	14/15 (93.3)	3/3 (100.0)	10/11 (90.9)
Positive antemortem HIV DNA PCR test[a]	22/24 (91.7)	–	1/1 (100.0)	–	12/13 (92.3)	1/1 (100.0)	8/9 (88.9)
Positive antemortem HIV antibody test[a]	18/21 (85.7)	–	4/5 (80.0)	–	6/7 (85.7)	3/3 (100.0)	5/6 (83.3)
Child had detectable viral load antemortem (RNA PCR)[a]	8/9 (88.9)	–	–	–	1/1 (100.0)	–	7/8 (87.5)
Child received ARV for treatment[a]	34/50 (68.0)	–	8/12 (66.7)	0/2 (0.0)	18/23 (78.3)	1/2 (50.0)	7/11 (63.6)
**HIV infected without malnutrition**	**56/141 (39.7)**	**1/1 (100.0)**	**13/36 (36.1)**	**2/9 (22.2)**	**10/35 (28.6)**	**8/27 (44.4)**	**18/33 (54.5)**
Any positive antemortem HIV test[a]	18/21 (85.7)	–	2/2 (100.0)	–	3/4 (75.0)	4/4 (100.0)	9/14 (81.8)
Positive antemortem HIV DNA PCR test[a]	13/15 (86.7)	–	1/1 (100.0)	–	1/1 (100.0)	3/3 (100.0)	8/10 (80.0)
Positive antemortem HIV antibody test[a]	12/13 (92.3)	–	2/2 (100.0)	–	3/4 (75.0)	4/4 (100.0)	3/3 (100.0)
Child had detectable viral load antemortem (RNA PCR)[a]	6/8 (75.0)	–	–	–	–	–	5/7 (71.4)
Child received ARV for treatment[a]	15/29 (51.7)	–	4/6 (66.7)	0/1 (0.0)	4/8 (50.0)	0/2 (0.0)	7/12 (58.3)

[a] Among deaths with antemortem clinical records regarding HIV testing and treatment.

eTable 5. Other causes of death in the causal chain for infant and child deaths with malnutrition as causal or significant condition by site, CHAMPS, 2016–2023 (N=632).

**Cause of death**	**Bangladesh** (N = 4) n (%)	**Ethiopia** (N = 78) n (%)	**Kenya** (N = 164) n (%)	**Mali** (N = 58) n (%)	**Mozambique** (N = 105) n (%)	**Sierra Leone** (N = 157) n (%)	**South Africa** (N = 66) n (%)
Lower respiratory infections	3 (75.0)	66 (84.6)	51 (31.1)	25 (43.1)	58 (55.2)	56 (35.7)	48 (72.7)
Sepsis	2 (50.0)	57 (73.1)	60 (36.6)	30 (51.7)	40 (38.1)	62 (39.5)	31 (47.0)
Diarrheal Diseases	2 (50.0)	35 (44.9)	21 (12.8)	21 (36.2)	25 (23.8)	35 (22.3)	10 (15.2)
Malaria	0 (0)	3 (3.8)	52 (31.7)	6 (10.3)	13 (12.4)	51 (32.5)	0 (0)
Anemias	0 (0)	4 (5.1)	3 (1.8)	1 (1.7)	5 (4.8)	95 (60.5)	0 (0)
HIV	0 (0)	0 (0)	23 (14.0)	7 (12.1)	25 (23.8)	15 (9.6)	14 (21.2)
Congenital birth defects	1 (25.0)	1 (1.3)	15 (9.1)	13 (22.4)	15 (14.3)	1 (0.6)	13 (19.7)
Meningitis/Encephalitis	0 (0)	19 (24.4)	2 (1.2)	8 (13.8)	1 (1.0)	6 (3.8)	7 (10.6)
Other infections	0 (0)	4 (5.1)	9 (5.5)	6 (10.3)	9 (8.6)	4 (2.5)	8 (12.1)
Other respiratory disease	0 (0)	3 (3.8)	6 (3.7)	1 (1.7)	7 (6.7)	7 (4.5)	6 (9.1)
Other	0 (0)	2 (2.6)	3 (1.8)	4 (6.9)	2 (1.9)	5 (3.2)	2 (3.0)
Tuberculosis	0 (0)	2 (2.6)	1 (0.6)	0 (0)	3 (2.9)	0 (0)	6 (9.1)
Other endocrine, metabolic, blood, and immune disorders	0 (0)	3 (3.8)	0 (0)	4 (6.9)	2 (1.9)	1 (0.6)	1 (1.5)
Other neurological disorders	0 (0)	0 (0)	2 (1.2)	5 (8.6)	2 (1.9)	0 (0)	1 (1.5)
Neonatal preterm birth complications	0 (0)	0 (0)	3 (1.8)	0 (0)	1 (1.0)	2 (1.3)	2 (3.0)
Liver disease	0 (0)	0 (0)	2 (1.2)	2 (3.4)	0 (0)	1 (0.6)	2 (3.0)
Injury	0 (0)	0 (0)	4 (2.4)	0 (0)	1 (1.0)	1 (0.6)	0 (0)
Measles	0 (0)	2 (2.6)	0 (0)	4 (6.9)	0 (0)	0 (0)	0 (0)
Other disorders of fluid, electrolyte and acid-base balance	0 (0)	1 (1.3)	2 (1.2)	1 (1.7)	0 (0)	0 (0)	2 (3.0)
Other immunodeficiencies	0 (0)	0 (0)	0 (0)	4 (6.9)	0 (0)	0 (0)	1 (1.5)
Other neonatal disorders	0 (0)	0 (0)	2 (1.2)	1 (1.7)	0 (0)	1 (0.6)	1 (1.5)
Sickle cell disorders	0 (0)	0 (0)	3 (1.8)	0 (0)	0 (0)	2 (1.3)	0 (0)
Kidney Disease	0 (0)	0 (0)	0 (0)	0 (0)	1 (1.0)	0 (0)	3 (4.5)
Other skin and subcutaneous diseases	0 (0)	0 (0)	2 (1.2)	0 (0)	1 (1.0)	0 (0)	1 (1.5)
Paralytic ileus and intestinal obstruction	0 (0)	0 (0)	2 (1.2)	1 (1.7)	1 (1.0)	0 (0)	0 (0)
Heart Diseases	0 (0)	0 (0)	1 (0.6)	1 (1.7)	0 (0)	0 (0)	1 (1.5)
Cancer	0 (0)	0 (0)	0 (0)	1 (1.7)	1 (1.0)	0 (0)	0 (0)
Congenital infection	0 (0)	1 (1.3)	0 (0)	0 (0)	0 (0)	0 (0)	1 (1.5)
Other gastrointestinal disease	0 (0)	0 (0)	1 (0.6)	1 (1.7)	0 (0)	0 (0)	0 (0)
Other nutritional deficiencies	0 (0)	1 (1.3)	0 (0)	1 (1.7)	0 (0)	0 (0)	0 (0)
Poisoning	0 (0)	0 (0)	0 (0)	0 (0)	1 (1.0)	1 (0.6)	0 (0)
Syphilis	0 (0)	0 (0)	1 (0.6)	0 (0)	1 (1.0)	0 (0)	0 (0)
Epilepsy	0 (0)	0 (0)	0 (0)	1 (1.7)	0 (0)	0 (0)	0 (0)
Perinatal asphyxia/hypoxia	0 (0)	0 (0)	0 (0)	1 (1.7)	0 (0)	0 (0)	0 (0)

eTable 6. Associations between individual measures of postmortem anthropometric malnutrition[a] and infectious causes[b] in the causal chain, CHAMPS, 2016–2023 (N = 1526[c]).

Variable	**Infectious cause of death** N = 1247 n (%)	**Non-infectious cause of death** N = 279 n (%)	**Crude Odds Ratio** (95% CI)	**P-value**	**Adjusted[d] Odds Ratio** (95% CI)	**P-value**
**Stunting**				<0.001		0.010
Yes	579 (46.4)	75 (26.9)	2.36 (1.78, 3.16)		1.56 (1.11, 2.19)	
No	668 (53.6)	204 (73.1)	*Reference*		*Reference*	
**Wasting**				<0.001		0.920
Yes	889 (71.3)	169 (60.6)	1.62 (1.23, 2.11)		0.98 (0.69, 1.40)	
No	358 (28.7)	110 (39.4)	*Reference*		*Reference*	
**Underweight**				<0.001		<0.001
Yes	805 (64.6)	120 (43.0)	2.41 (1.86, 3.15)		2.04 (1.41, 2.95)	
No	442 (35.4)	159 (57.0)	*Reference*		*Reference*	
**HIV infection[e]**						
Yes	124 (9.9)	15 (5.4)	1.94 (1.15, 3.51)	0.018	1.59 (0.90, 2.82)	0.110
No	1123 (90.1)	264 (94.6)	*Reference*		*Reference*	

[a] Stunting: LAZ<-2, wasting: WLZ<-2 or MUACZ<-2, underweight: WAZ<-2.
[b] Infectious causes of death include diarrheal diseases, congenital infection, lower respiratory infections, malaria, measles, meningitis/encephalitis, other infections, rabies, sepsis, syphilis, tuberculosis, and upper respiratory infections
[c] This is a complete case analysis excluding deaths missing anthropometric values or with implausible anthropometric measures for stunting, wasting, or underweight.
[d] Adjusted for the other variables listed in the model as well as age group, sex, location of death, and site as a random effect. Variance inflation factors were <1.4 for all independent variables. Hosmer-Lemeshow C statistic = 11.011, p-value = 0.201. Hosmer-Lemeshow H statistic = 5.670, p-value = 0.684.
[e] Interaction terms between HIV infection and stunting (p=0.111), wasting (p=0.674), and underweight (p=0.511) were not statistically significant.

eTable 7. Associations between combinations of postmortem anthropometric malnutrition measures[a] and infectious causes[b] in the causal chain, CHAMPS, 2016–2023 (N = 1526[c]).

Variable	**Infectious cause of death** N = 1247 n (%)	**Non-infectious cause of death** N = 279 n (%)	**Crude Odds Ratio** (95% CI)	**Adjusted[d] Odds Ratio** (95% CI)
Underweight only	15 (1.2)	1 (0.4)	5.47 (1.08, 99.54)	5.58 (0.72, 43.26)
Wasting only	128 (10.3)	53 (19.0)	0.88 (0.59, 1.32)	0.93 (0.62, 1.41)
Stunting only	48 (3.8)	8 (2.9)	2.19 (1.05, 5.15)	1.95 (0.88, 4.35)
Underweight & wasting	278 (22.3)	60 (21.5)	1.69 (1.17, 2.45)	1.85 (1.26, 2.71)
Underweight & stunting	48 (3.8)	11 (3.9)	1.59 (0.82, 3.35)	1.44 (0.70, 2.92)
Wasting & stunting	19 (1.5)	8 (2.9)	0.87 (0.38, 2.16)	0.79 (0.33, 1.93)
Underweight, wasting & stunting	464 (37.2)	48 (17.2)	3.52 (2.41, 5.20)	3.51 (2.33, 5.27)
Normal	247 (19.8)	90 (32.3)	*Reference*	*Reference*

[a] Stunting: LAZ<-2, wasting: WLZ<-2 or MUACZ<-2, underweight: WAZ<-2; normal: LAZ≥-2 & WLZ≥-2 & WAZ≥-2 & MUACZ≥-2.
[b] Infectious causes of death include diarrheal diseases, congenital infection, lower respiratory infections, malaria, measles, meningitis/encephalitis, other infections, rabies, sepsis, syphilis, tuberculosis, and upper respiratory infections
[c] This is a complete case analysis excluding deaths missing anthropometric values or with implausible anthropometric measures for stunting, wasting, or underweight.
[d] Adjusted for the other variables listed in the model as well as age group, sex, location of death, HIV infection, and site as a random effect. Variance inflation factors were <1.1 for all independent variables. Hosmer-Lemeshow C statistic = 10.238, p-value = 0.249. Hosmer-Lemeshow H statistic = 12.641, p-value = 0.125.

eTable 8. Positive TAC PCR tests by specimen for 109 deaths with malnutrition as causal or significant condition attributed to *Escherichia coli/Shigella.*

Specimen	Test	N (%)
Blood	*Escherichia coli/Shigella*	63 (57.8)
CSF	*Escherichia coli/Shigella*	19 (17.4)
Rectal swab	*Escherichia coli* (aaiC gene)	45 (41.3)
Rectal swab	*Escherichia coli* (aatA gene)	62 (56.9)
Rectal swab	*Escherichia coli* (bfpA gene)	26 (23.9)
Rectal swab	*Escherichia coli* (eae gene)	16 (14.7)
Rectal swab	*Escherichia coli* (heat-labile enterotoxin)	11 (10.1)
Rectal swab	*Escherichia coli* (heat-stable enterotoxin)	29 (26.6)
Rectal swab	*Escherichia coli/Shigella* (ipaH gene)	19 (17.4)

TAC: TaqMan Array Card; PCR: polymerase chain reaction

eFigure 1. Flowchart of enrolled under-five infant and child deaths from CHAMPS sites between December 2016 – December 2023, that had minimally invasive tissue samples (MITS) and consent only for verbal autopsy and clinical abstraction (non-MITS) and included in the analysis.

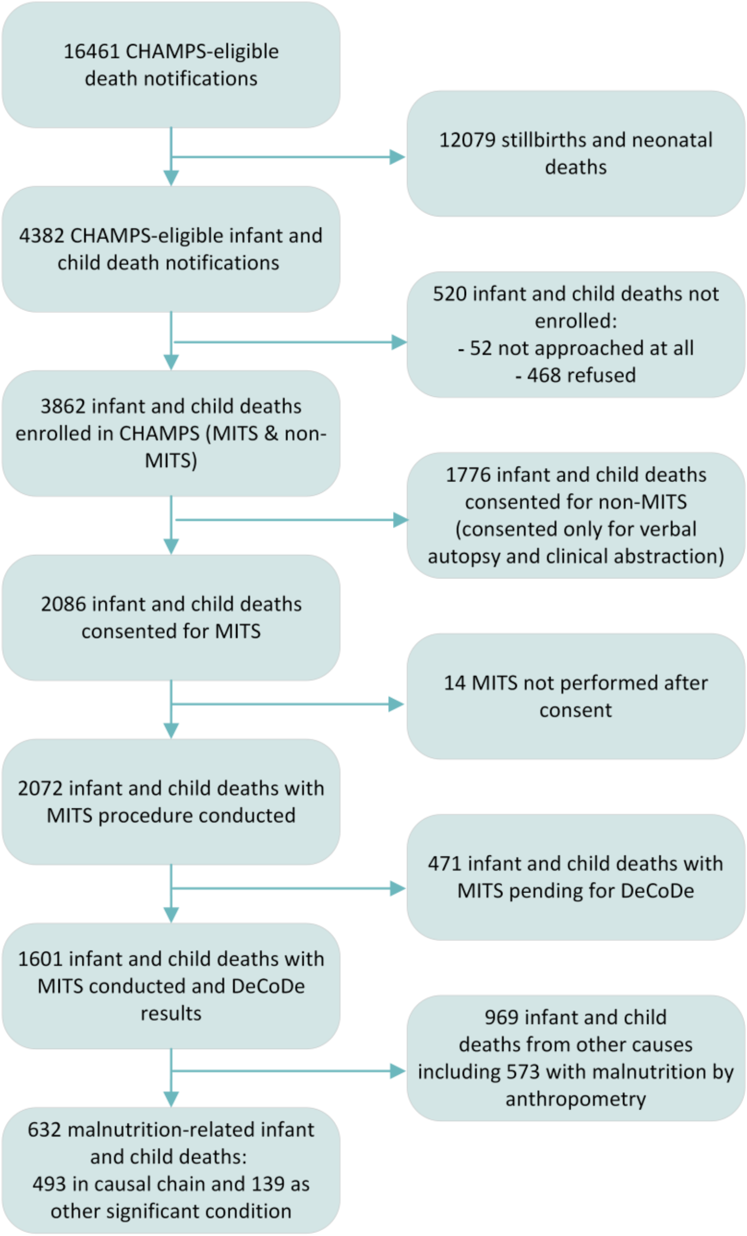

eFigure 2. Flowchart of enrolled under-five infant and child deaths from CHAMPS sites between December 2016 – December 2023, that had minimally invasive tissue samples (MITS) and consent only for verbal autopsy and clinical abstraction (non-MITS) and included in the analysis by site.

	Bangladesh	Ethiopia	Kenya	Mali	Mozambique	Sierra Leone	South Africa
CHAMPS-eligible death notifications	4335	2904	1619	1771	2387	1549	2051
CHAMPS-eligible infant and child death notifications	456	843	711	490	686	686	510
Infant and child deaths enrolled in CHAMPS (MITS & non-MITS)	272	799	630	487	671	582	421
Infant and child deaths consented for MITS	16	137	562	168	297	511	395
Infant and child deaths with MITS procedure conducted	16	135	559	168	294	506	394
Infant and child deaths with MITS conducted and DeCoDe results	10	89	379	133	261	375	354
Malnutrition-related infant and child deaths	4	78	164	58	105	157	66

eFigure 3. Anthropometric measures for infant and child deaths with malnutrition as causal or significant condition, CHAMPS, 2016–2023 (N=632).

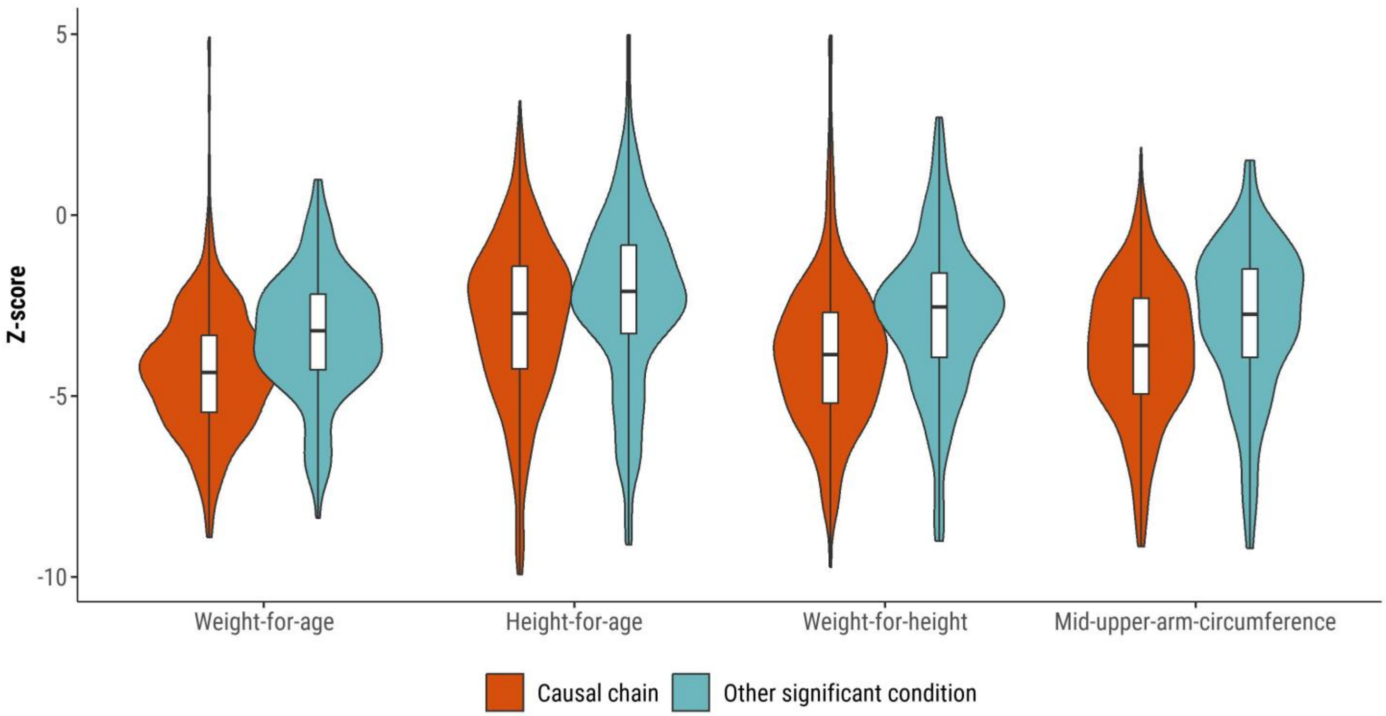

eFigure 4. Venn diagram of the proportion of deaths with malnutrition as causal or significant condition who were wasted (weight-for-height Z-score < -2 or mid-upper arm circumference Z-score < -2), stunted (height-for-age Z-score < -2), or underweight (weight-for-age Z-score < -2), CHAMPS, 2016–2023 (N=632).

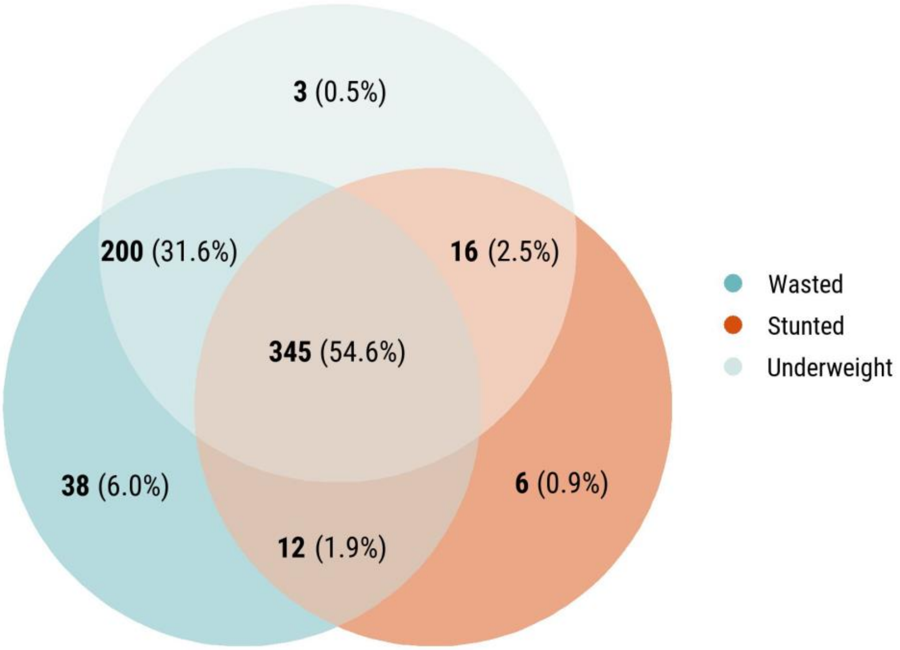

eFigure 5. Other causes of death (A) and pathogens (B) when malnutrition is a causal or significant condition, CHAMPS, 2016–2023 (N=632).

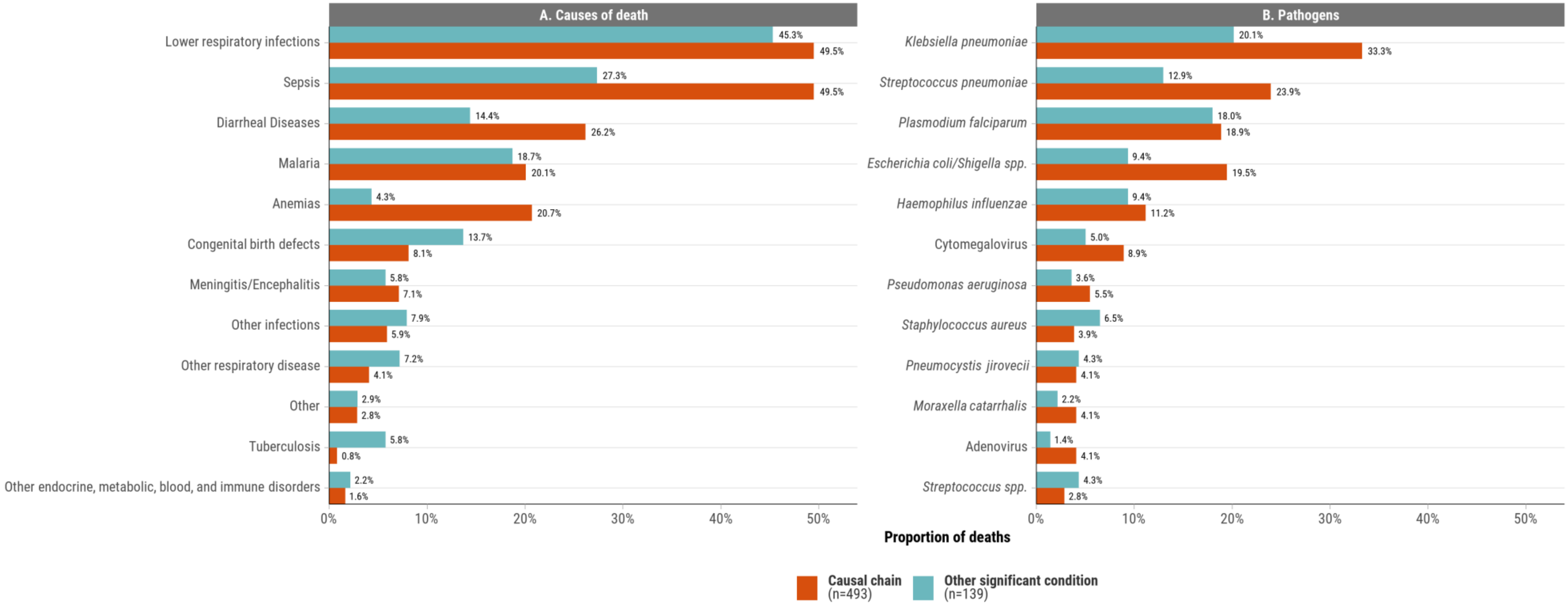

eFigure 6. Underlying causes of death (A) and pathogens (B) when malnutrition is the antecedent/immediate cause of death (red), and antecedent/immediate causes of death and pathogens when malnutrition is the underlying cause of death (blue), CHAMPS, 2016–2023 (N=493).

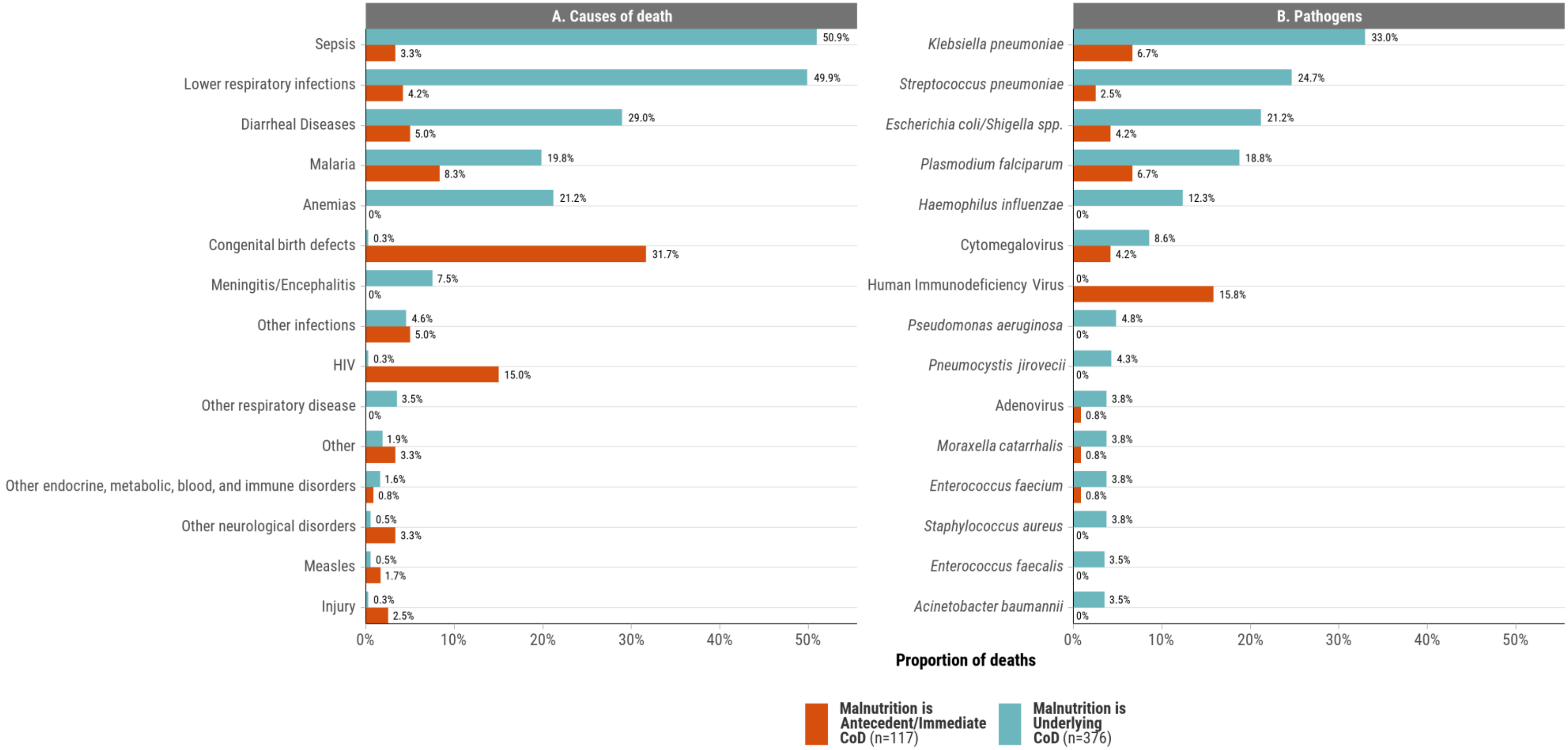

eFigure 7. Boxplots of anthropometric measures for infant and child deaths with infectious and non-infectious causes of death, CHAMPS, 2016–2023 (N=1601).

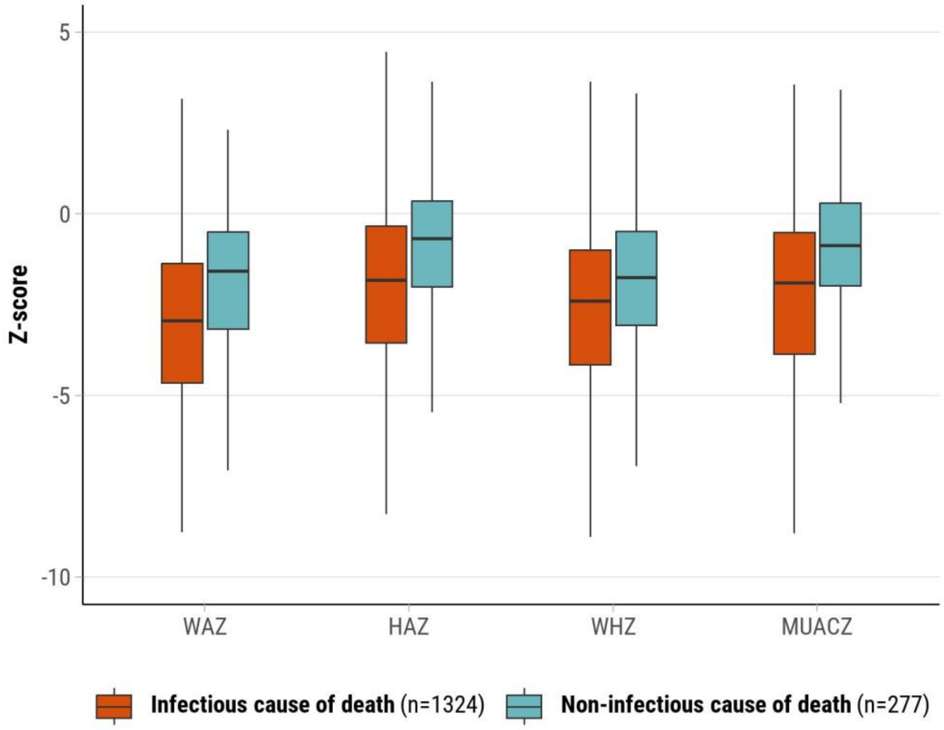

Infectious causes of death include diarrheal diseases, congenital infection, lower respiratory infections, malaria, measles, meningitis/encephalitis, other infections, rabies, sepsis, syphilis, tuberculosis, and upper respiratory infections.

eFigure 8. Unadjusted and adjusted[a] odds of a death having malnutrition as causal or significant condition for the most frequent causes of death in the causal chain among infant and child deaths, CHAMPS, 2016–2023 (N=1601).

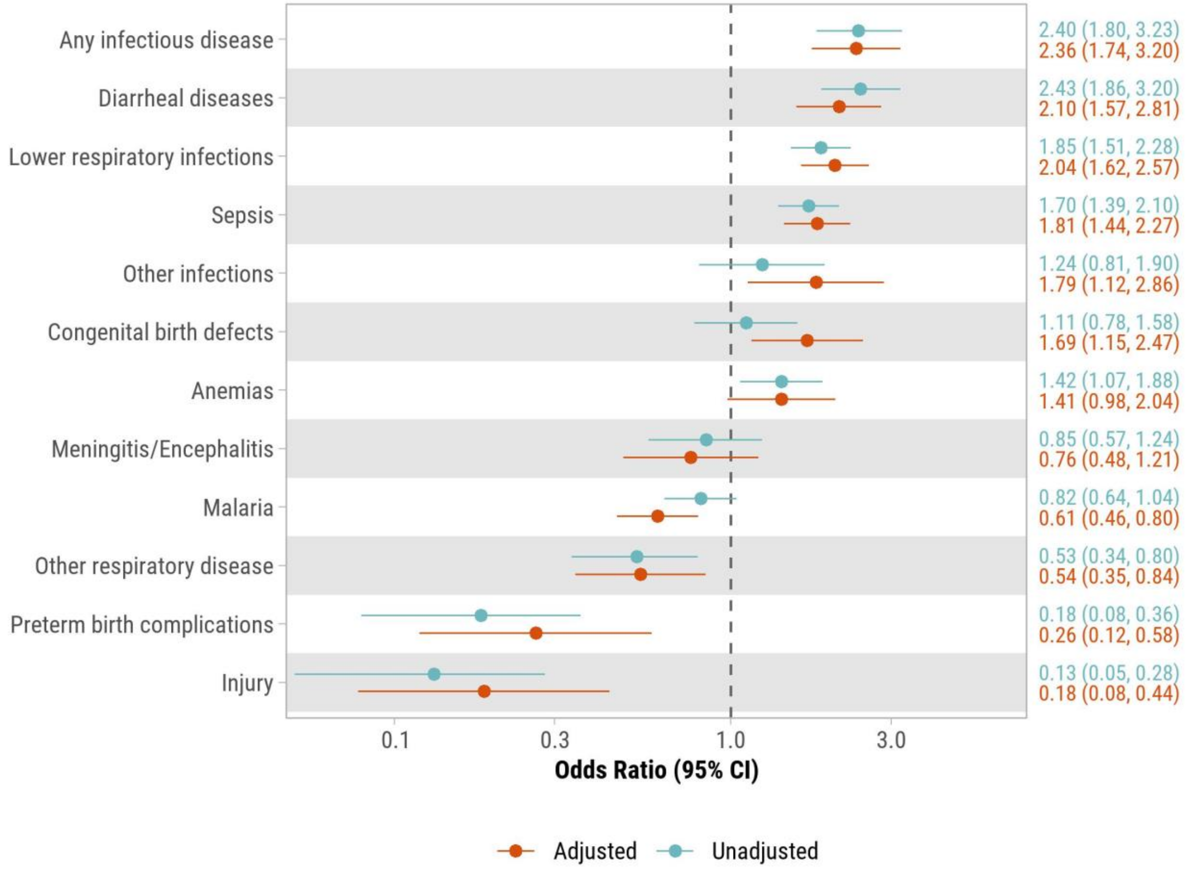

The x-axis is shown on a $\log_{10}$ scale. Odds ratios and 95% confidence intervals are shown.

[a] Adjusted for age group, sex, location of death, and site as a random effect. 'Any infectious disease' includes: diarrheal diseases, congenital infection, lower respiratory infections, malaria, measles, meningitis/encephalitis, other infections, rabies, sepsis, syphilis, tuberculosis, and upper respiratory infections. The association between each cause of death and malnutrition were from separate regression models. The reference groups are all deaths from non-infectious causes (top row), non-sepsis deaths (fourth row), etc.

eFigure 9. Frequencies of co-infections among infant and child deaths with malnutrition as causal or other significant condition, CHAMPS, 2016–2023 (N = 632).

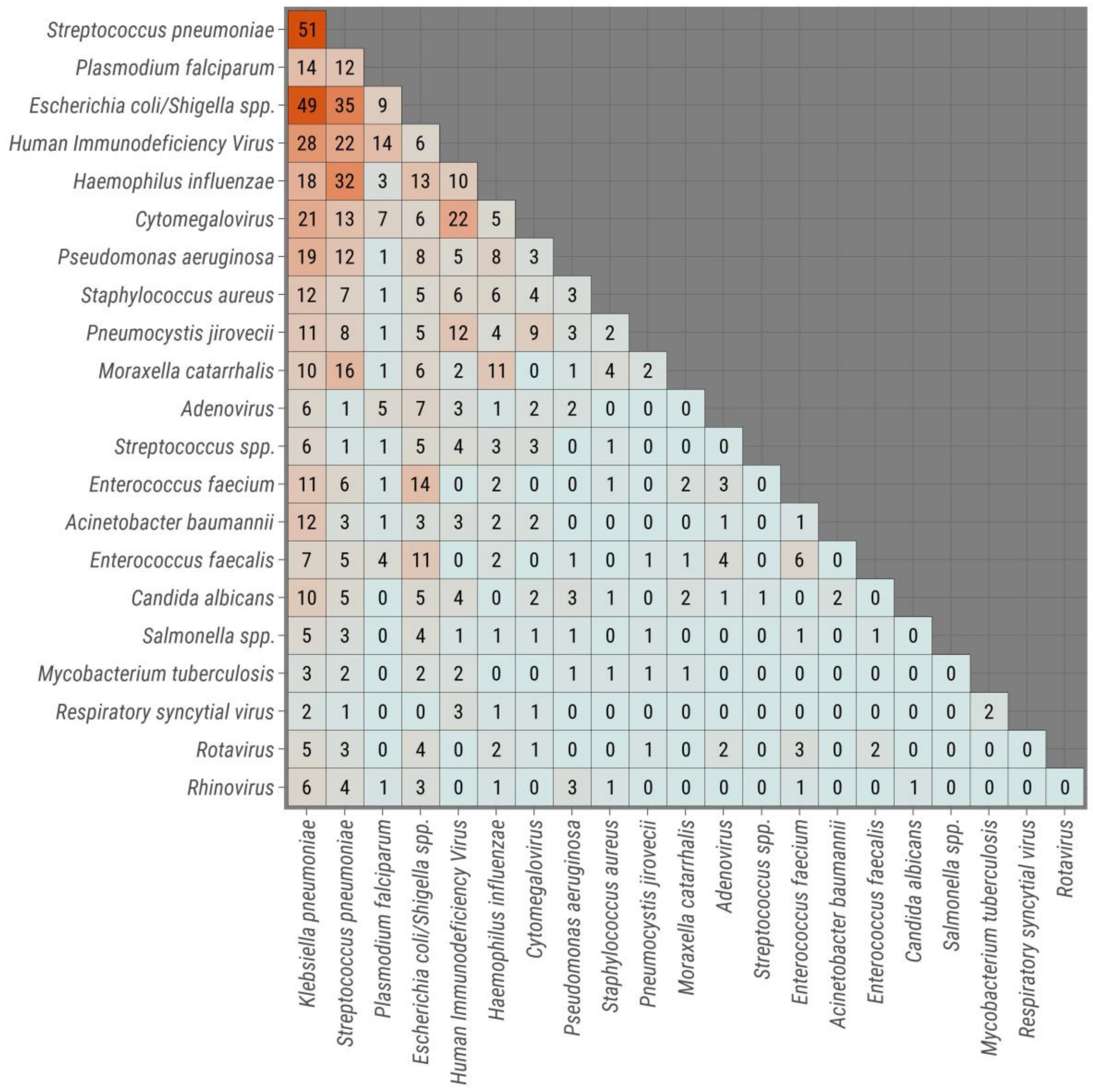

eFigure 10. Expert (DeCoDe) panel recommendations for preventing deaths with malnutrition as causal or other significant condition, CHAMPS, 2016–2023 (N = 526[a]).

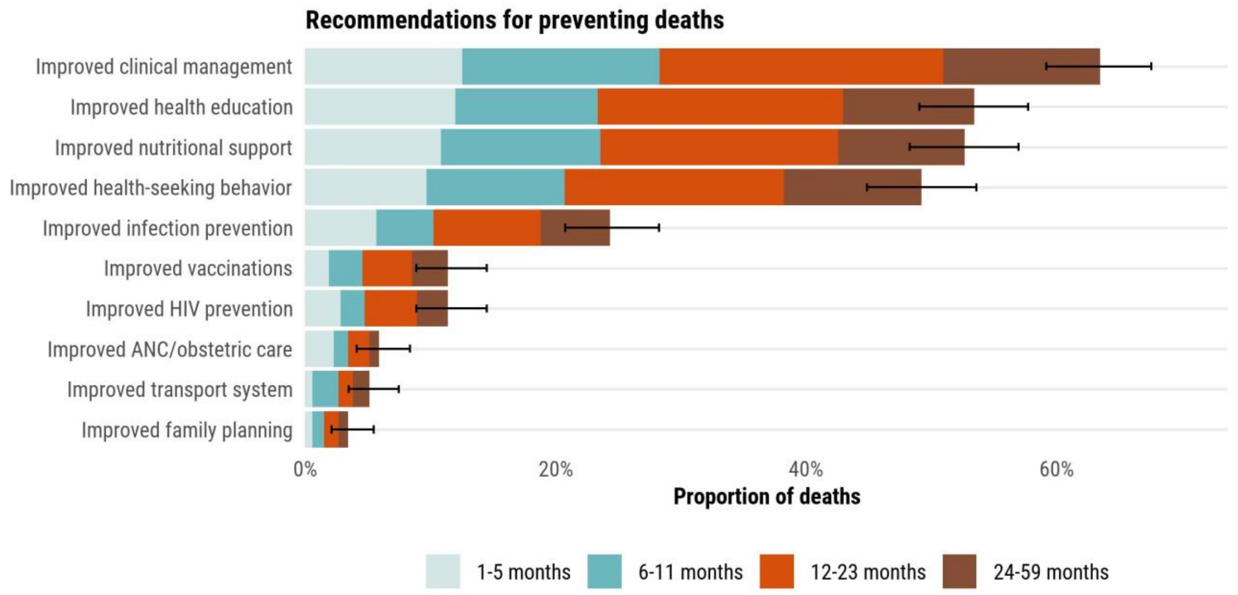

[a] Of 632 deaths with malnutrition, 567 were deemed preventable by the expert DeCoDe panel. Recommendations were provided for 526 of the preventable deaths.

References

1. Salzberg NT, Sivalogan K, Bassat Q, et al. Mortality surveillance methods to identify and characterize deaths in child health and mortality prevention surveillance network sites. *Clinical Infectious Diseases*. 2019;69(Supplement_4):S262-S273.
2. Taylor AW, Blau DM, Bassat Q, et al. Initial findings from a novel population-based child mortality surveillance approach: a descriptive study. *The Lancet Global Health*. 2020;8(7):e909-e919.
3. Blau DM, Caneer JP, Philipsborn RP, et al. Overview and development of the child health and mortality prevention surveillance determination of cause of death (decode) process and decode diagnosis standards. *Clinical Infectious Diseases*. 2019;69(Supplement_4):S333-S341.
4. World Health Organization. Verbal autopsy standards: the 2012 WHO verbal autopsy instrument. *Geneva: WHO*. 2012;
5. World Health Organization. *The International Statistical Classification of Diseases and Health Related Problems ICD-10: Tenth Revision. Volume 1: Tabular List*. vol 1. World Health Organization; 2004.
6. Breiman RF, Blau DM, Mutevedzi P, et al. Postmortem investigations and identification of multiple causes of child deaths: An analysis of findings from the Child Health and Mortality Prevention Surveillance (CHAMPS) network. *PLoS Medicine*. 2021;18(9):e1003814.
7. Madewell ZJ, Whitney CG, Velaphi S, et al. Prioritizing Health Care Strategies to Reduce Childhood Mortality. *JAMA Netw Open*. Oct 3 2022;5(10):e2237689. doi:10.1001/jamanetworkopen.2022.37689
8. Paganelli CR, Kassebaum N, Strong K, et al. Guidance for Systematic Integration of Undernutrition in Attributing Cause of Death in Children. *Clin Infect Dis*. Dec 15 2021;73(Suppl_5):S374-s381. doi:10.1093/cid/ciab851
